# Cancer‐associated fibroblast‐derived exosomal microRNA‐20a suppresses the PTEN/PI3K‐AKT pathway to promote the progression and chemoresistance of non‐small cell lung cancer

**DOI:** 10.1002/ctm2.989

**Published:** 2022-07-20

**Authors:** Lin Shi, Weiliang Zhu, Yuanyuan Huang, Lin Zhuo, Siyun Wang, Shaobing Chen, Bei Zhang, Bin Ke

**Affiliations:** ^1^ Department of Traditional Chinese Medicine Zhujiang Hospital of Southern Medical University Guangzhou China; ^2^ Department of Cancer Center Zhujiang Hospital of Southern Medical University Guangzhou China; ^3^ Department of VIP Region State Key Laboratory of Oncology in South China Collaborative Innovation Center of Cancer Medicine Sun Yat‐sen University Cancer Center Guangzhou China

**Keywords:** CAF, exosome, microRNA‐20a, NSCLC, PTEN

## Abstract

**Background:**

Cancer‐associated fibroblasts (CAFs) contributes to overall tumor progression. In the current survey, we explored the ability of microRNA‐20a (miR‐20a) within these CAF‐derived exosomes to influence non‐small‐cell lung cancer (NSCLC) progression.

**Materials and methods:**

Normal tissue‐associated fibroblasts (NAFs) and CAFs were collected from samples of NSCLC patient tumors and paracancerous lung tissues. Exosomes derived from these cells were then characterized via Western blotting, nanoparticle tracking analyses, and transmission electron microscopy. The expression of miR‐20a was assessed via qPCR and fluorescence in situ hybridization (FISH). CCK‐8, EdU uptake, and colony formation assessments were used for evaluating tumor proliferation, while Hoechst staining was performed to monitor the in vitro apoptotic death of tumor cells. A model of xenograft tumor established in nude mice was also used to evaluate in vivo tumor responses.

**Results:**

CAF‐derived exosomes exhibited miR‐20a upregulation and promoted NSCLC cell proliferation and resistance to cisplatin (DDP). Mechanistically, CAF‐derived exosomes were discovered to transmit miR‐20a to tumor cells wherein it was able to target PTEN to enhance DDP resistance and proliferation. Associated PTEN downregulation following exosome‐derived miR‐20a treatment enhanced PI3K/AKT pathway activation.

**Conclusion:**

The achieved outcomes explain that CAFs can release miR‐20a‐containing exosomes capable of promoting NSCLC progression and chemoresistance, highlighting this pathway as a possible therapeutic target in NSCLC.

## Graphical abstract

1. CAFs‐derived exosomal miR‐20a promotes the progression of NSCLC.
2. CAFs‐derived exosomal miR‐20a promotes chemoresistance of NSCLC.
3. PTEN is a target gene of miR‐20a. 4. miRNA‐20a suppresses the PTEN/PI3K‐AKT pathway.

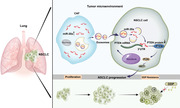

## 1 INTRODUCTION

Lung carcinoma is a form of malignancy with extremely considerable incidence and mortality rates among both men and women. Despite both of these rates having risen over the past half‐century, there is still a pressing need for the development of more robust and reliable treatments for affected patients.^1^, ^2^ Non‐small‐cell lung cancer (NSCLC) cases account for an estimated 85% of total cases of lung carcinoma,^3^ and platinum‐based chemotherapies remain the standard of care for individuals with advanced stage disease.^4^ At present, the frequent emergence of chemoresistance represents a major barrier to substantial improvements in patient prognosis, underscoring the need for more in‐depth analyses of the mechanisms governing such drug resistance in an effort to better define approaches to effectively treating this type of cancer.

The tumour microenvironment (TME) consists of tumour cells, proximal stromal and immune cells and the secreted factors produced by both of these cellular populations. In many cases, tumours can deplete local immune‐related cell populations or induce fibroblasts to produce pro‐oncogenic signals.^5^, ^6^, ^7^ For example, cancer‐associated fibroblasts (CAFs) can release pro‐angiogenic factors capable of promoting tumour growth and angiogenesis.^8^, ^9^ CAFs are proliferative and metabolically active cells with a fibroblast phenotype that can be involved in all processes of cancer progression.^8^ It has been reported that the higher the number of CAFs infiltrated in the TME of lung cancer patients, the worse the clinical prognosis.^10^ As the depth of research regarding the interplay between non‐coding RNAs and macromolecular transfer between cells grows, several researchers have posited that CAFS can communicate with nearby tumour cells in a manner conducive to tumour growth by releasing microRNAs (miRNAs) encapsulated within exosomal vesicles.^11^, ^12^, ^13^

Exosomes are small (40–120 nm) membrane‐enclosed vesicles that encapsulate a range of macromolecules such as proteins, DNA and RNA.^14^, ^15^ These exosomes are secreted through an extensive range of cell types and are present within biofluids including the blood, urine, saliva and cerebrospinal fluid wherein they can facilitate intercellular communication. Exosomes contain specific characteristic protein antigens including Alix, TSG101, integrins, Rab GTPases and tetraspanins (CD63, CD9, CD81 and CD82).^16^, ^17^ As the macromolecules enclosed within exosomes are shielded from efficient degradation, they can effectively facilitate the transfer of information and signal transduction molecules between local cell populations or distant anatomical locations.^18^ Some reports have highlighted key functional roles for exosomes in the context of lung cancer development.^19^, ^20^ miRNAs are a kind of short non‐coding RNAs that suppress target mRNA at the posttranscriptional level through binding to the complementary sequences within their 3′‐ UTR sequences and destabilizing these mRNAs or preventing their translation.^21^, ^22^ Recent research suggests that TAM‐derived exosomes can transfer miR‐21 to gastric carcinoma cells, thereby conferring resistance to cisplatin (DDP).^23^ CAFs created from patient‐derived pancreatic cancer tumour samples were innately chemoresistant and played positive roles in promoting multiplication and chemotherapy resistance of pancreatic carcinoma cells through exosome signalling.^24^ MiRNA assay results revealed that miR‐20a were observably up‐regulated in CAFs compared with normal fibroblasts (NFs), and CAFs promoted lung cancer cell growth and migration.^25^ MiRNA‐20a plays as potential both oncogene and tumour suppressor gene. Study also signified that up‐regulation of miR‐20a facilitated breast cancer growth and chemoresistance through regulating MAPK1/c‐Myc pathway.^26^ Nevertheless, suppression of miR‐20a expression promoted HL60 and K562 cell apoptosis and changed chemoresistance in K562/ADR cell lines.^27^ Whether CAFs‐derived exosomal miRNAs, particularly miR‐20a contributes to emergence of NSCLC chemoresistance remains unestablished.

Here, we initially extracted CAF‐ and normal tissue‐associated fibroblast (NAF)‐derived exosomes from samples of cell‐conditioned media (CM), and we then demonstrated that CAFs were readily internalized by NSCLC cells. We first determined that miR‐20a was most substantially expressed in CAF‐derived exosomes. Besides, we first proved that miR‐20a could be efficiently transferred from CAFs into NSCLC cells through an exosome‐mediated mechanism, thereby influencing the the functions of NSCLC cells, especially DDP resistance. Mechanistically, we further verified that miR‐20a was able to promote DDP resistance and proliferation via targeting phosphatase and tensin homolog (PTEN) and promoting PI3K/AKT pathway activation in NSCLC cells. Other research has verified that miR‐20a can promote NSCLC proliferation by up‐regulating programmed cell death‐ligand 1 (PD‑L1) and inhibiting PTEN.^28^ However, the target‐regulatory relationship between miR‐20a and PTEN is unclear, and the effect of both on NSCLC cell chemoresistance has not been reported. Overall, our data presented that exosomal miR‐20a might represent a novel CAF‐derived oncogenic factor that can contribute to the onset and progression of this malignant disease.

## 2 MATERIALS AND METHODS

### 2.1 Patients’ tissues and ethics statement

A total of 90 pairs non‐samll cell lung carcinoma tissues and pericarcinomatous tissues were obtained during surgery from patients diagnosed with NSCLC who had not undergone neoadjuvant chemotherapy or prior radiotherapy treatment at the Sun Yat‐sen University Cancer Center. For primary NAFs and CAFs isolation, tissue samples were collected and used for primary cell isolation in State Key Laboratory of Oncology in South China within 1 h. Other collected tissue samples were frozen in liquid nitrogen immediately and stored at −80°C until use. The Ethics Committee of Sun Yat‐sen University Cancer Center confirmed the present exploration (number: GZR2020‐297), with all participants having presented written informed consent before enrollment.

### 2.2 Fluorescence in situ hybridization

A fluorescence in situ hybridization (FISH) Kit (RiboBio, Guangzhou, China) was employed in line with the provided directions. Briefly, cells were rinsed by implementing phosphate buffered saline (PBS), fixed for 10 min with 4% formaldehyde, and permeabilized in .5% Triton X‐100 at 4°C for 5 min. Subsequently, cells were rinsed thrice with PBS, pre‐hybridized at 37°C in a RNA‐FISH Hybridization System (Novobiotec, Beijing, China) for 30 min, and next incubated overnight with an anti‐miR‐20a oligodeoxynucleotide probe at 37°C while protected from light. Cells were then counterstained using DAPI and imaged with a confocal laser‐scanning microscope (Carl Zeiss).

### 2.3 Patient‐derived fibroblast isolation and culture

Primary NAFs and CAFs were collected from NSCLC tissue specimens from cases experiencing lobectomy, with CAFs being harvested from the center of tumours with maximal diameters <10 mm, while NAFs were collected from normal tissue regions at least 10 cm from the tumour margins based on previous studies.^25^, ^29^ Briefly, the samples of tissue were minced into 1–3 mm^3^ fragments and digested with Dulbecco’s modified Eagle’s medium (DMEM) (HyClone, UT, USA) including 300 units/ml collagenase (Sigma‐aldrich; Cat. No. SCR103), and 100 units/ml hyaluronidase (Merck KGaA, Darmstadt, Germany; Cat. No. H1115000), 10 ng/ml Choleragen (MedChemExpress, Shanghai, China) and 2% BSA (Promega, USA) at 37°C for 2 h with constant agitation. Cells were subsequently accumulated via centrifugation (1000 × g for 5 min), cleaned wich PBS, and resuspended in dulbecco's modified eagle medium comprising 10% fetal bovine serum (FBS), passed through a cell strainer with 100 μm diameter, plated in 60‐mm tissue cultivation plates in DMEM/F12 (Life Technologies, USA) comprising 100 U/ml penicillin (Servicebio, Wuhan, China), 100 mg/ml streptomycin (Servicebio, Wuhan, China), 10% FBS (Sigma, MO, USA) and 5 μg/ml insulin (MedChemExpress, Shanghai, China) and cultured in a 37°C 5% CO_2_ humidified incubator. The CM was changed twice a week. After three passages, exosomes were extracted. The subtypes of CAFs were identified by vimentin and ASMA to ensure repeatability. CM was prepared by incubating fibroblasts for 2 days in DMEM supplemented with 10% exosome‐free FBS, after which media was collected, spun for 5 min at 4°C, 1000 × g and 30 min at 4°C, 10 000 × g, and passed through a filter with .22‐μm diameter. In co‐culture assays, NAFs or CAFs were combined with NSCLC cells at a 3:1 ratio, with tumour cells initially being added to 6‐well plates after which CAFs/NAFs were added to the upper of the Boyden chamber (Corning, cat no. 3413) with .4 μm pore sizes.

### 2.4 Exosome isolation and fluorescent labeling

Differential centrifugation was used to isolate exosomes from CM samples as in previous research.^30^ Briefly, cell culture media was initially centrifuged at 300 × g for 10 min, and the supernatant was further centrifuged 2000 × g for 20 min, and finally the supernatant was centrifuged at 10 000 × g for 30 min at 4°C to eliminate cellular debris, succeeded by ultracentrifugation twice for 90 min at 100 000 × g to pellet exosomes, with PBS being used to resuspend the exosomal pellet between spins. The nanoparticle tracking analysis (NTA) was performed to characterized the isolated exosomes using a ZetaView PMX 110 instrument (Particle Metrix, Germany), followed by use in subsequent experiments. And also detected with transmission electron microscopy analysis as described previously.^31^

A PKH67 green fluorescent labeling kit (Sigma) was employed based on provided directions to label isolated exosomes as in prior reports.^32^ Briefly, exosomes were harvested and were suspended in diluent C (total 100 μl) and combined with PKH67 dye solution (100 μl, 4 × 10^−6^ M). Following a 5‐min incubation, the labeling reaction was terminated via the addition of serum (200 μl). Labelled exosomes were subsequently rinsed two times using PBS and combined with differently treated NSCLC cells for 24 h, with a microscope being utilized to monitor the uptake of these labelled particles.

### 2.5 Cell culture and transfection

A549, H838, HCC827 and H1299 NSCLC cells were obtained from the ATCC and characterized using short tandem repeat markers (Genetic Testing Biotechnology Corporation, Suzhou, China). The cells were cultivated in DMEM comprising penicillin‐streptomycin (Servicebio, Wuhan, China) and 10% FBS in a 5% CO_2_ incubator at 37°C. The cisplatin‐resistant A549 and H1299 cell lines were established by ourselves and stored in our laboratory.^33^, ^34^ Cisplatin‐resistant cells were cultured in complete medium supplemented with 1 μg/ml DDP. Lipofectamine 2000 (Invitrogen, USA) was employed to transfect these cells based on provided directions.

### 2.6 Plasmid, inhibitor and mimic constructs

The PTEN cDNA sequence was cloned and connected into the pcDNA3.1‐Myc/His expression vector (Invitrogen), with sequencing being used to confirm accurate construct construction. In luciferase reporter assays, the 3′‐UTR of PTEN wild type (WT) sequence was cloned and connected into the pmiRGLO vector (Promega, WI, USA), with site‐directed mutagenesis additionally being used to generate a mutated (MUT) version of this reporter vector. miR‐20a mimics, inhibitors, PTEN siRNAs (5′‐GCAGCCGTTCGGAGGATTATT‐3′ and 5′‐GTCAACAACTTACACTTATTT‐3′) and corresponding control constructs were obtained from Shanghai GenePharma (China).

### 2.7 Immunofluorescent staining

Fibroblasts were grown on coverslips, cells were fixed using 4% paraformaldehyde for 10 min, after washed with PBS, cells were permeabilized using .1% Triton X‐100, then blocked using 3% goat serum (Wolcavi, Beijing, China), and incubated at 4°C by utilizing primary anti‐α‐SMA (abcam, ab124964, 1:500), anti‐fibronection (abcam, ab2413, 1:300) or anti‐vimentin (abcam, ab8978, 1 μg/ml). Samples were then probed with AF488 or AF647‐conjugated anti‐rabbit or anti‐mouse IgG (abcam, 1:500) for 1 h at ambient temperature, protected from light, following which DAPI (Invitrogen, USA, 1:300) was employed for nuclear counterstaining.

### 2.8 Western blotting

A total protein extraction kit (Bio‐Rad, CA, USA) was employed for protein extraction, following which equal amounts of protein (30 μg/sample) were divided through sodium dodecyl sulfate‐polyacrylamide gel Electrophoresis and transferred onto polyvinylidene fluoride membranes (Millipore, MA, USA). Blots were blocked implementing 5% non‐fat milk, incubated during the night hours at 4°C with antibodies specific for CD63 (abcam, ab216130, 1:800); CD81 (abcam, ab155760, 1:1000), TSG101 (abcam, ab125011, 1: 2000), PI3K (abcam, ab32089, 1:1000), PI3K‐p85 (phospho Y607) (abcam, ab182651, 1:1000), PI3K‐p110 (abcam, ab151549, 1:2000), AKT (Invitrogen, AHO1112, 1:1200), AKT (phospho T308) (ab38449, 1:1000), PTEN (abcam, ab170941, 1:1500), CD31 (abcam, ab281583, 1:1000), CD45 (abcam, ab10558, 1:1000) and 66 kDa (proteintceh, 16848‐1‐AP, 1:1000), α‐smooth muscle actin (α‐SMA) (abcam, ab124964, 1:1500), S100A4, S100 calcium binding protein A4 (FSP1) (S100A4, abcam, ab197896, 1:1200), Fibronection (abcam, 285285, 1:1200), platelet derived growth factor receptor beta (PDFGRβ) (abcam, ab69506, 1:800), Vimentin (abcam, ab8978, 1:2000), FAP (abcam, ab207178, 1:1100), alpha Tubulin (abcam, ab7291, 1:5000). Blots were then probed using horseradish peroxidase (HRP)‐conjugated anti‐rabbit or anti‐mouse secondary antibodies (1:3000, ZhongShanJinQiao, BeiJing, China), followed by blot development using an enhanced chemiluminescence reagent.

### 2.9 qPCR

TRIzol reagent (Invitrogen, USA) was employed for the extraction of RNA from cells, after which a reverse transcriptase kit (TaKaRa, Dalian, China) was implemented for the preparation of cDNA. miRNA first‐strand cDNA was constructed using the stem‐loop method^35^ using the cDNA Synthesis Kit (R601, Novabio, Shanghai, China). Stem‐loop primer 5′‐CTCAACTGGTGTCGTGGAGTCGGCAATTCAGTTGAGCTACCTGC‐3′ was purchased from General Biol (Anhui, China). The realtime fluorescence quantitative polymerase chain reaction (qPCR) reactions were executed with SYBR Green Premix ExTaq II (TaKaRa), with β‐actin serving as an internal reference. The 2^−ΔΔCt^ approach was employed to evaluate relative gene expressions. Primers were as follows: PTEN‐forward: 5′‐TGGATTCGACTTAGACTTGACCT‐3′ and PTEN‐reverse: 5′‐GGTGGGTTATGGTCTTCAAAAGG‐3′; β‐actin‐forward: 5′‐CATGTACGTTGCTATCCAGGC‐3′ and β‐actin‐reverse: 5′‐CTCCTTAATGTCACGCACGAT‐3′. miR‐20a‐forward: 5′‐ACACTCCAGCTGGGTAAAGTGCTTATAGTGCAG‐3′ and miR‐20a‐reverse: 5′‐CTCAACTGGTGTCGTGGA‐3′; U6‐forward: 5′‐CTCGCTTCGGCAGCACA‐3′ and U6‐reverse: 5′‐AACGCTTCACGAATTTGCGT‐3′. U6 was as a reference control for miR‐20a.

Exosome concentrations were measured via BCA and NTA assays, with 10 μg (2 × 10^8^) exosomes or 1 × 10^6^ treated cells being utilized to extract RNA for miRNA analyses. A total of .5 μg of extracted RNA was then employed to prepare cDNA with a Mir‐X miRNA First‐Strand Synthesis kit, after which qPCR analyses were performed as above using U6^36^ or miR‐16^37^ as normalization controls.

### 2.10 Cell proliferation assay and colony formation assays

A CCK‐8 kit (Dojindo, Japan) was employed for the assessment of cellular proliferation. Briefly, cells were added to the plates containing 96 wells (2 × 10^3^/well) at 37°C for 2 h, with absorbance then being assessed on three consecutive days at 450 nm.

In the case of the colony formation assessments, the treated cells were cultured for 14 days and were fixed using methanol at 20–25°C for 10 min, then cells were stained using .1% crystal violet (Sigma) for 15 min at 37°C, and counted via microscopy.

### 2.11 IC50 calculations

The values of half‐maximal inhibitory concentration (IC50) for DDP were measured by plating cells in 96‐well plates for 24 h and subsequently replacing culture milieu with milieu comprising DDP (0, .39, .78, 1.56, 3.125, 6.25, 12.5, 25 or 50 μg/ml) for 48 h. CCK‐8 reagent was subsequently added for an additional 24 h, after which supernatants were removed, and DMSO was added to each well. Next, by implementing a microplate reader, absorbance at 490 nm was measured (Bio‐Rad, USA). The specimens were scrutinized in triplicate.

### 2.12 EdU uptake assay

An EdU assessment kit (Ribobio, Guangzhou, China) was employed for the assessment of cellular multiplication. Briefly, cells were added to confocal plates (10 × 10^5^ /well) and incubated for 2 h with EdU buffer (50 μM) at 37°C, followed by fixation for 30 min with 4% formaldehyde and permeabilization with .1% Triton X‐100 for 20 min. Following the addition of the EdU solution to these cells, Hoechst was used for nuclear staining, and cells were visualized via fluorescence microscopy.

### 2.13 Hoechst 33342 assay

Cell apoptosis was measured using Hoechst 33342 staining (ThermoFisher, USA) according to the specification as previously reported.^33^ A fluorescence microscope (Leica, Cat. #DMI6000B) was implemented for visualizing the frequency of apoptotic cells.

### 2.14 Predition of miR‐20a target genes

The possible target genes of miR‐20a were predicted through DIANA‐microT,^38^ miRanda (http://www.microrna.org/) with score <−.5, PicTar (http://www.pictar.org/) and TargetScanHuman8.0 (www.targetscan.org/vert_80/) with score <−.2 databases.

### 2.15 Gene ontology and pathway analysis

Gene ontology (GO) analysis was applied to understand the biological processes (BPs), molecular function (MF) and cell component (CC) of gene enrichment.^39^ Kyoto Encyclopedia of Genes and Genomes (KEGG) pathway analysis was applied to analyze genomic, chemical, and systemic functional information of the enriched genes.^40^ DAVID (http://www.david.abcc.ncifcrf.gov/) was used to visualize the enrichment of genes in BPs, MF and CC and pathway (*p* < .05).^41^, ^42^

### 2.16 Luciferase reporter assays

Both pmirGLO plasmids harbouring WT or mutated (MUT) versions of the PTEN 3′‐UTR and miRNA mimics were co‐transfected into 293T cells with Lipofectamine 2000 (Invitrogen). Following a 48 h incubation, a Dual‐Luciferase Reporter Assay Kit (Promega) was employed in line with the presented directions to assess luciferase activity, with Renilla luciferase activity being implemented for normalization.

### 2.17 RNA immunoprecipitation

A commercial RNA‐binding protein immunoprecipitation kit (Millipore, USA) was employed to conduct RNA immunoprecipitation (RIP) assays based on provided directions and relevant research.^43^ The specificity of precipitated RNA species was assessed using IgG and total RNA (*n* = 3) as controls. For anti‐AGO2 RIP assays, miRNA mimics were initially transfected into appropriate NSCLC cells and then an anti‐AGO2 antibody (2 μg/ml, abcam, clone number: ab32381) was employed 48 h post‐transfection. Primers were designed as follows: PTEN‐forward: 5′‐GGACATTTAAAATTCAATTAG‐3′ and PTEN‐reverse: 5′‐ACACATCAGTCTGTCTCCAC‐3′.

### 2.18 Xenograft tumour modelling

A total of 84 female BALB/c nude mice (4‐week‐old) were separated into fourteen groups at random (*n* = 6/group) for use in studies conducted as in prior reports.^44^ CAFs stably expressing miR‐20a were obtained using a lentiviral vector with geneticin selection. Mice received a subcutaneous flank injection of 3 × 10^6^ HCC827 cells combined (1:1) with NAF, CAF or CAF/shRab27a cells in a total 200 μl volume of PBS and basement membrane matrix (Corning, England; Cat. No. 354248) by a ratio of 1:1.^45^ Additional, 3 × 10^6^ HCC827 cells and 100 μg of exosomes derived from NAFs and CAFs were hypodermically injected into the left flank of nude mice,^46^ and 100 μg of exosomes derived from NAFs and CAFs were injected into the subcutaneous tumour once a week. When the subcutaneous tumour grew to about 5 mm in diameter, 4 mg/kg DDP was injected intraperitoneally into nude mice once every 3 days for six consecutive times.^47^ When tumours had grown sufficiently to be visible to the naked eye, tumour growth was monitored once per week with Vernier calipers. The volume of the tumour was measured as follows: volume = .5 × length × width^2^. Following a 28‐day period, mice were killed, and xenografts were collected for the next assessments. This study was consistent with the Helsinki Declaration. The NIH Guide for the Care and Use of Laboratory Animals was used when designing all animal studies (number: 2019–075).

### 2.19 Immunohistochemical staining

Referring to previous research,^48^ the xenograft tumours were first fixed in 4% paraformaldehyde, dehydrated with gradient alcohol, transparent with xylene at room temperature and embedded in paraffin. Then the paraffin‐embedded tissues were sectioned to 4‐μm thick slices. Then the slices were deparaffinized, roasted at 56°C for 2 h and treated with xylene. After antigen repair, the slices of each group were incubated overnight with Ki‐67 antibody (abcam, ab16667; 1:200) at 4°C, followed by 2 h incubation with peroxisase‐bound secondary antibody. Diaminobenzine colourization was performed using the EnVision Detection System Kit (DAKO, Denmark), followed by nuclear staining with haematoxylin. After washing, the cells were imaged via brightfield microscopy (Olympus, Japan).

### 2.20 Statistical analyses

Data were performed in thrice and were displayed as the mean ± standard deviation (SD) and analysed employing PASW Statistics 18.0 (IBM, SPSS, IL, USA) and GraphPad Prism 9.0 (GraphPad computer program, CA, USA), and were compared via Student's *t*‐tests or one‐way analysis of variance (ANOVAs) followed by Tukey's or Dunnett's post hoc tests, respectively. χ^2^ ‐tests were used to measure the relationship between miR‐20a expression levels and clinical pathologic features of NSCLC patients. Pearson correlation coefficient was used for analyzing the relationship between miR‐20a and PTEN expression. *p* < .05 was the threshold of significance.

## 3 RESULTS

### 3.1 CAF‐derived exosomes exhibit miR‐20a up‐regulation

**FIGURE 1:**
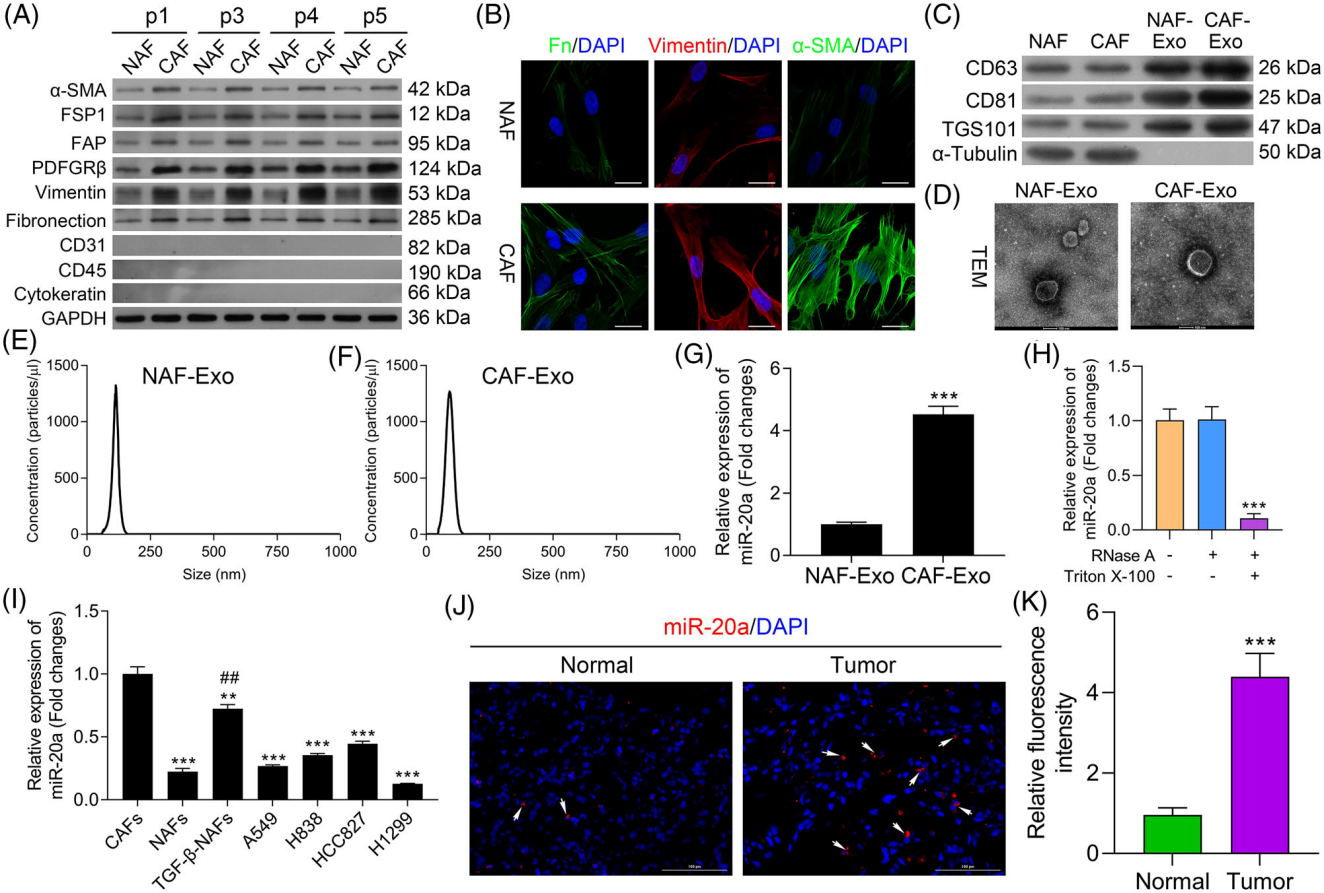
Exosomes derived from cancer‐associated fibroblasts (CAFs) exhibit miR‐20a upregulation. (A) α‐SMA, FSP1, PDFGRβ, vimentin, fibronectin, CD31, CD45 and cytokeratin levels were measured via Western blotting in CAFs and normal tissue‐associated fibroblasts (NAFs). (B) α‐SMA, vimentin and fibronectin levels were assessed by immunofluorescent staining in the 1st, 3rd, 4th and 5th generations of CAFs and NAFs. (C) CD63, CD81 and TSG101 levels were measured via Western blotting in CAFs, NAFS and exosomes derived from these cells. (D) CAF‐ or NAF‐condition media‐derived exosomes were analyzed via electron microscopy (scale bar, 100 nm). (E and F) Vesicles derived from CAF‐ or NAF‐conditioned media were analyzed via a nanoparticle tracking analysis. (G) Relative miR‐20a expression in exosomes derived from CAFs and NAFs. (H) After treatment with 2 mg/ml RNase alone or combined with .1% Triton X‐100, miR‐20a level was analysed using qPCR in the culture medium of CAFs. (I) miR‐20a expression levels in NAFs, TGF‐β‐NAFs and A549, H838 and HCC827 cells were assessed via qPCR. (J) miR‐20a expression was assessed by fluorescence in situ hybridization (FISH) in non‐small‐cell lung cancer (NSCLC) samples and healthy pulmonary tissue. (K) Quantitative analysis of relative fluorescence intensity. Results are representative data from triplicate experiments, and outcomes are means ± standard deviation (SD) (**p < .01, ***p < .001 vs. CAFs; ^##^ *p*< .01 vs. NAFs)

We began by collecting NAFs and CAFs from healthy paracancerous lung tissues and NSCLC tumours, respectively, with both cell populations exhibiting vimentin positivity and spindle‐like morphology. Western blotting data first revealed that relative to NAFs, α‐SMA, FSP1, PDFGRβ, vimentin and fibronectin expressions were markedly elevated in CAFs, and which was not significantly changed in the 1, 3, 4 and 5 generations of CAFs; besides, we discovered that CD31, CD45 and cytokeratin proteins were not detected in both NAFs and CAFs (Figure 1A). Relative to NAFs, CAFs expressed greater levels of fibroblast marker proteins including vimentin, fibronectin, and α‐smooth muscle actin (α‐SMA) (Figure 1B). Exosomes are small membrane‐enclosed particles that can be released by fibroblasts and other cell types, facilitating intercellular communication within the TME.^49^ We subsequently harvested exosomes from CAF‐ and NAF‐derived CM via ultracentrifugation. Western blotting analyses of these exosomes revealed them to express higher levels of exosomal marker proteins (TSG101, CD63 and CD81) relative to levels in CAFs and NAFs, with no α‐tubulin being evident therein (Figure 1C), in line with prior reports regarding exosomes.^50^ In subsequent qNano and electron microscopic analyses, these particles were confirmed to be 80–120 nm in diameter with a double‐layered membrane (Figure 1D–F). Overall, these results confirmed that exosomes had been successfully isolated.

Exosomes frequently encapsulate large quantities of miRNAs, facilitating their transportation between cells. In prior reports, miR‐20a was shown to be highly up‐regulated in CAFs and linked to tumour chemoresistance.^25^ As such, we sought to discover the expression of miR‐20a within CAF‐ and NAF‐derived exosomes, revealing it to be significantly up‐regulated within the exosomes harvested from CAFs (Figure 1G). Meanwhile, we explored the presence of extracellular miR‐20a. It was found that processing with RNase did not alter the level of miR‐20a in culture medium, but combined treatment of Triton X‐100 and RNase significantly eliminated the level of miR‐20a, which demonstrated that extracellular miR‐20a was prevailingly wrapped by the membrane rather than directly released (Figure 1H). We then evaluated the expression of this miRNA in NAFs and in the A549, H838, HCC827 and H1299 NSCLC cell lines, revealing it to be significantly down‐regulated therein, whereas it was up‐regulated in TGF‐β treated NAFs (TGF‐β‐NAFs) relative to the levels in NAFs (Figure 1I). We further measured its expression in NSCLC patient tissue samples via FISH, revealing it to be up‐regulated in NSCLC tissue samples relative to healthy paracancerous pulmonary tissue (Figure 1J,K). Together, these data suggest that CAF‐derived exosomes exhibit miR‐20a up‐regulation.

### 3.2 GW4968 and Rab27a silencing down‐regulate miR‐20a in HCC827 and H1299 cells

**FIGURE 2:**
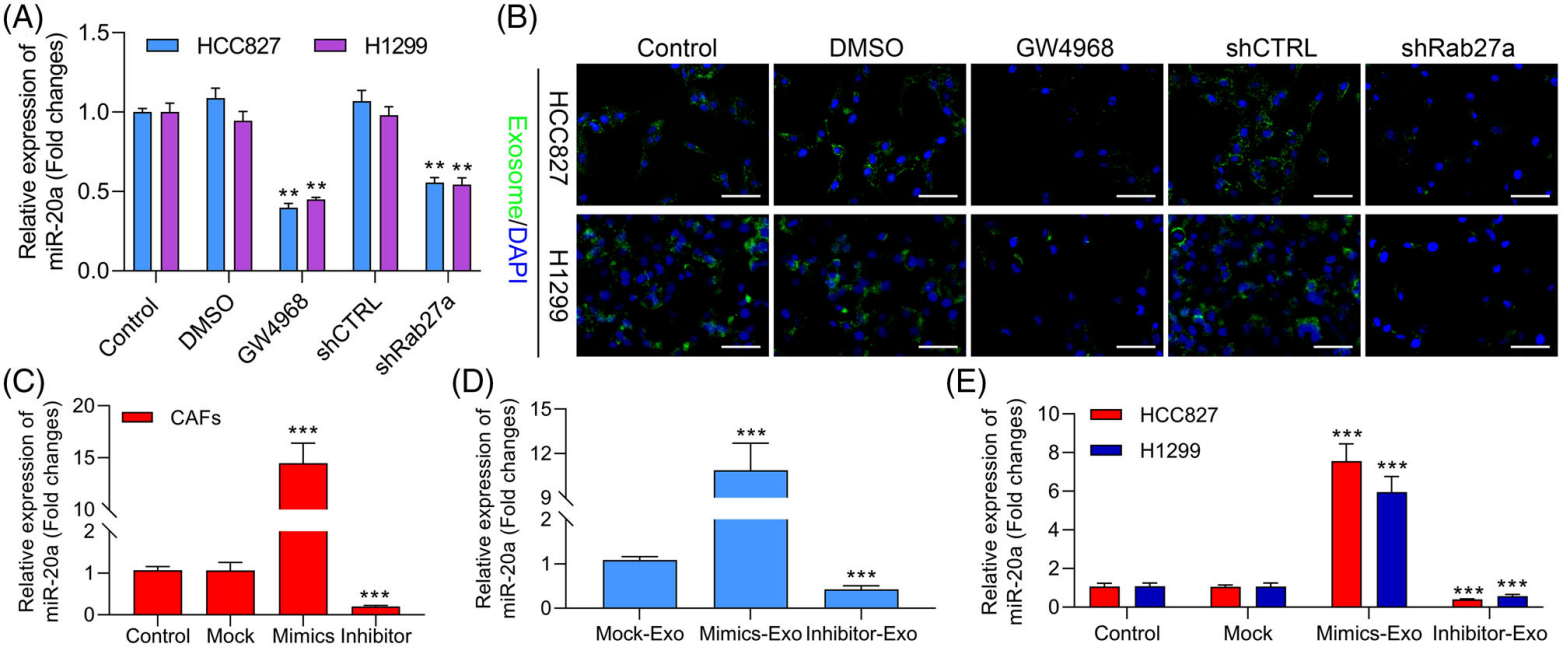
GW4968 and Rab27a silencing down‐regulate miR‐20a in HCC827and H1299 cells. (A) Following co‐culture with cancer‐associated fibroblast (CAF)‐derived exosomes treated with GW4869 or shRab27a, miR‐20a expression was analyzed in HCC827 and H1299 cells. (B) PKH67‐labelled exosomal uptake by the treated HCC827 and H1299 cells, with representative images being shown. (C) miR‐20a levels were measured via qPCR in CAFs following miR‐20a mimic or inhibitor treatment. ***p < .001 versus Control. (D) Relative expression of miR‐20a was assessed via qPCR in exosomes derived from CAFs, which were transfected with miR‐20a mimic or inhibitor. (E) After transfection with miR‐20a mimic or inhibitor in CAFs, CAF‐derived exosomes were collected and used to treat HCC827 or H1299 cells, with miR‐20a expression in these cells then being assessed via qPCR. ***p < .001 versus Control

To further determine which miRNAs were contained within CAF‐derived exosomes and thereby delivered to NSCLC cells, we next treated HCC827 and H1299 cells with exosomes harvested from NAFs or CAFs and then assessed the expression of potential miRNAa via qPCR. As shown in Figure S1A, the expressions of miR‐20a, miR‐210, miR‐103a‐3p, miR‐369 and miR‐130a were markedly elevated in CAF‐derived exosomes relative to that in NAF‐derived exosomes, and miR‐20a was most up‐regulated in CAF‐derived exosomes among these five miRNAs. Thus, we chose miR‐20a for further study. Also, we screened the optimal treatment concentration of exosome inhibitor (GW4869) in CAFs and NAFs. CCK‐8 data revealed that GW4869 did not affect the viviability of CAFs and NAFs (Figure S1B). And our data uncovered that GW4869 obviously reduced the concentration of exosomes in CAFs and NAFs, especially 20 and 50 μM GW4869 (Figure S1C). In our next study, we chose 20 μM GW4869 to treat CAFs. Meanwhile, modification of the Rab27a gene has been reported to regulate the efficacy of exosome secretion and action.^51^ Thus, we treated CAFs with GW4869 or shRab27a for 24 h to block exosome production reduced the levels of miR‐20a in both of these cell lines upon subsequent CAF‐exosome treatment (Figure 2A), indicating that CAF‐derived miRNAs are primarily delivered to NSCLC cells through exosome‐mediated mechanisms. We then used the PKH26 dye to monitor CAF‐derived exosome uptake by these NSCLC cells. At 3 h post‐treatment, red fluorescence was visible within both tested cell lines consistent with efficient exosomal uptake (Figure 2B), thus confirming that CAFs can secrete exosomes, which can subsequently be internalized by HCC827 and H1299 cells. We then measured levels of miR‐20a within CAFs following miR‐20a mimic or inhibitor treatment, revealing that miR‐20a mimic treatment markedly enhanced the expression of this miRNA within these cells (Figure 2C). The expression levels of miR‐20a within exosomes derived from these CAFs were also assessed following miR‐20a mimic or inhibitor treatment (Figure 2D). Next, exosomes were used to treat HCC827 and H1299 cells in culture. Subsequent qPCR analyses revealed that exosomes derived from CAFs tranfected with miR‐20a mimic, and inhibitor increased and reduced the expressions of miR‐20a, respectively, (Figures 2E), consistent with the capability of CAF‐released exosomes to transfer this miRNA to NSCLC cells.

### 3.3 miR‐20a‐containing CAF‐released exosomes promote NSCLC cell multiplication, DDP resistance and apoptotic inhibition

**FIGURE 3:**
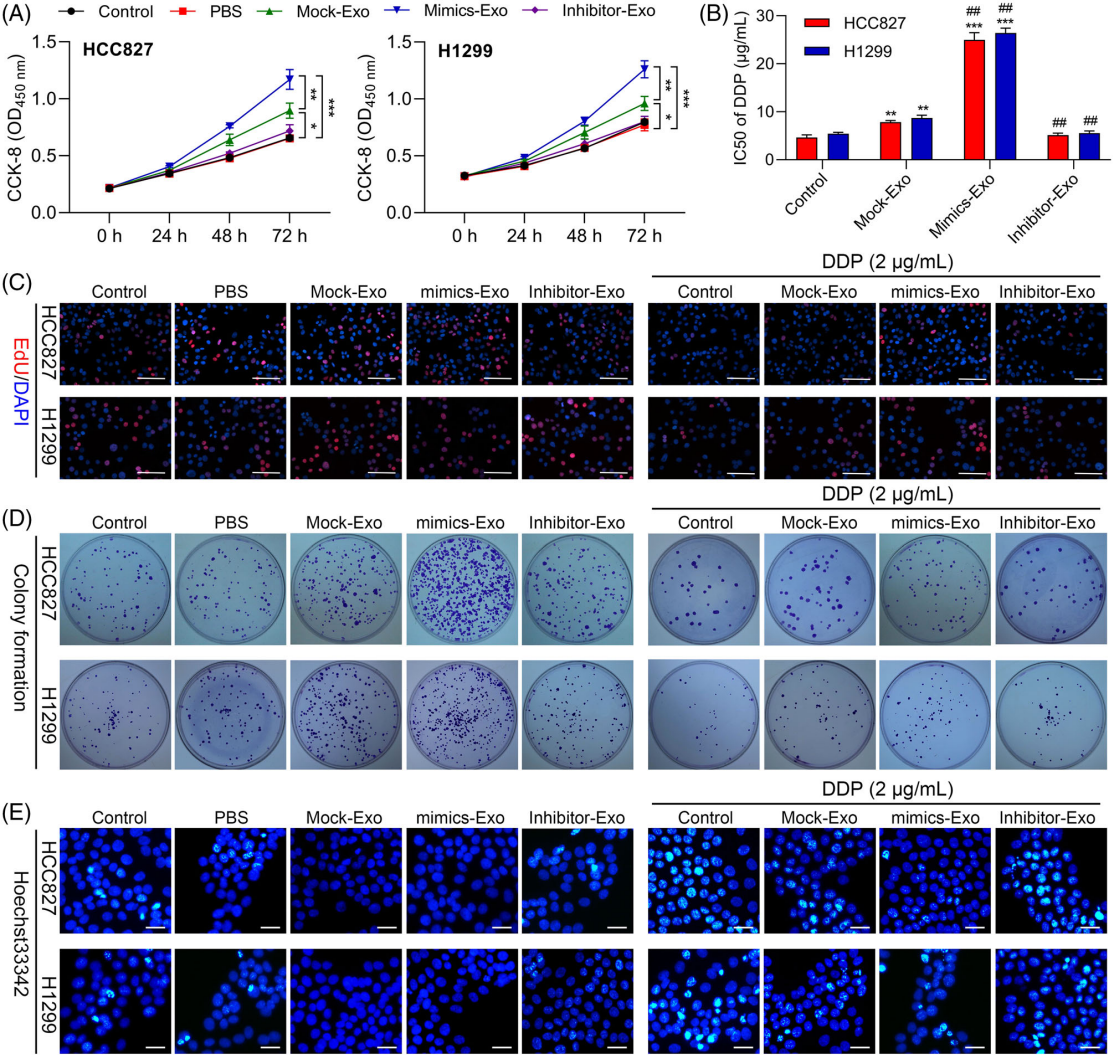
Non‐small‐cell lung cancer (NSCLC) patient‐derived cancer‐associated fibroblasts (CAFs) promote exosome‐mediated proliferation and DDP resistance in NSCLC cells. (A) CCK‐8 assessment was implemented to assess cellular proliferation, *p < .05, **p < .01, ***p < .001. (B) CCK‐8 assessment was used to calculate DDP IC50 values for HCC827 and H1299 cells. **p < .01, ***p < .001 versus control; ^##^ *p* < .01 versus mock‐exosome. The effects of miR‐20a mimic treatment on NSCLC cell proliferation, apoptosis and DDP resistance as measured via EdU assay (scale bar, 50 μm) (C), colony formation assay (D) and Hoechst staining (E). Results are representative data from triplicate experiments, and data are means ± standard deviation (SD)

Next, we further testified that Exo‐miR‐20a mimics were further found to enhance the colony formation and proliferation activity of both NSCLC cell lines while suppressing their apoptotic death (*p* < .05), whereas the opposite was observed following exo‐miR‐20a inhibitor treatment (Figure 3A,C,D and Figure S2A,B). A CCK‐8 assessment was additionally employed to appraise the effects of DDP treatment on these cells, revealing that exo‐miR‐20a mimic treatment led to a substantial increase in the DDP IC50 value (Figure 3B and Table 1). To more fully explore the degree of DDP resistance in HCC827 and H1299 cells, both cell lines were treated for 24 h with this chemotherapeutic drug (2 μg/ml), after which EdU uptake, colony formation and Hoechst staining assays were performed (Figure 3C,D,E and Figure S2B,C). The proliferative rate of cells co‐cultured with exosomes transfected with miR‐20a mimics rose significantly with a concomitant drop in apoptotic induction, whereas the opposite trend was observed following miR‐20a inhibitor treatment, indicating that miR‐20a‐containing CAF‐derived exosomes can induce DDP resistance within NSCLC cells. Together, these results thus suggest that these miR‐20a‐enriched exosomes harvested from CAFs can enhance NSCLC cell proliferative activity and chemoresistance while suppressing the induction of apoptosis.

**TABLE 1:** IC50 of DDP in HCC828 and H1299 cells co‐cultured with exosome

	HCC827 (μg/ml)	H1299 (μg/ml)
Control	4.67 ± .54	5.45 ± .26
Mock‐Exo	7.84 ± .34^**^	8.73 ± .56^**^
Mimics‐Exo	24.97 ± 1.51^[Link]^, ^[Link]^	26.42 ± 1.04^[Link]^, ^[Link]^
Inhibitor‐Exo	5.15 ± .38^##^	5.56 ± .46^##^

Abbreviation: DDP, Cisplatin.

^**^ *p* < .01.

^***^ *p* < .001 versus Control.

^##^ *p* < .01 versus Mock‐Exosome.

### 3.4 miR‐20a induces proliferation and DDP resistance in NSCLC cells in vitro

**FIGURE 4:**
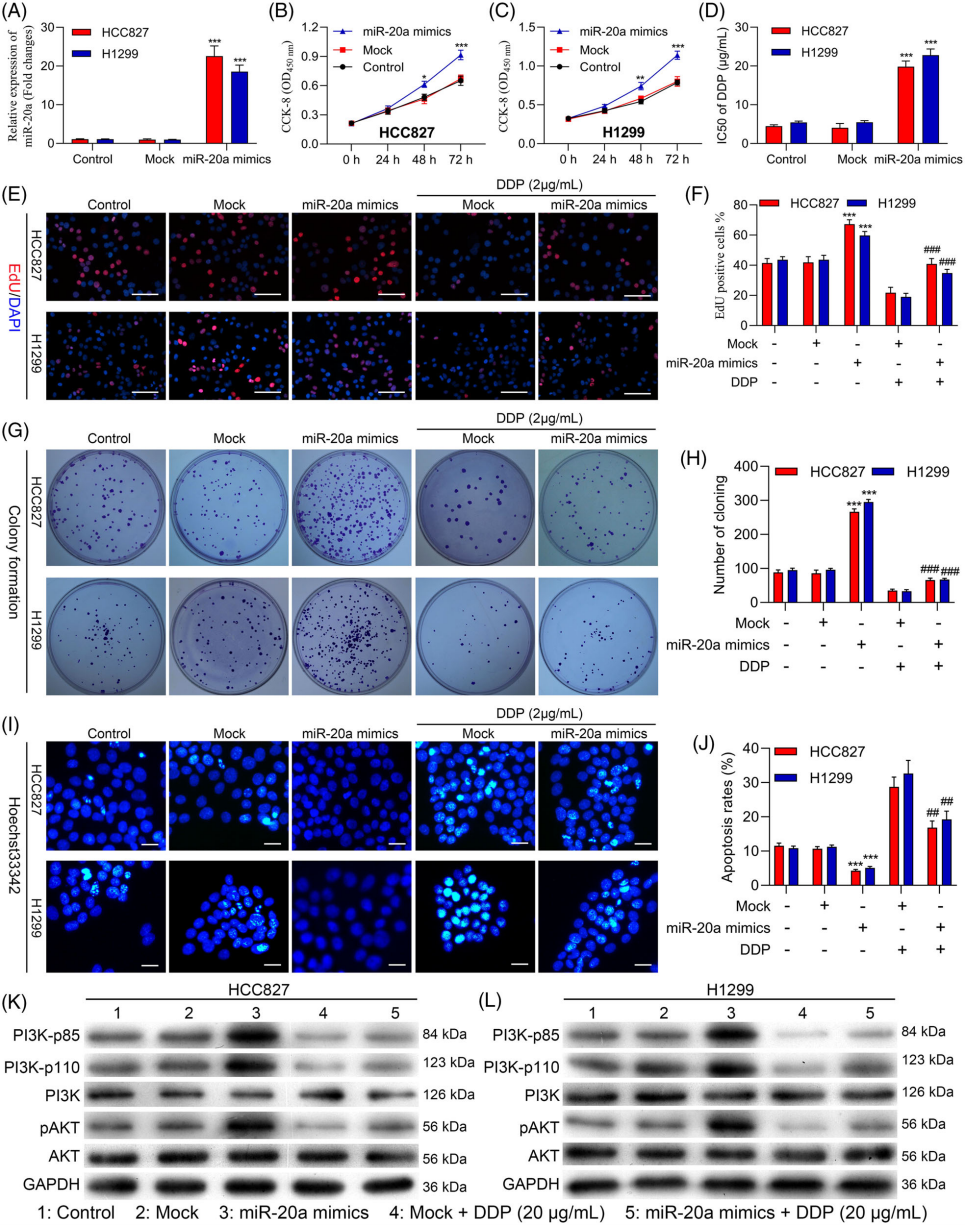
miR‐20a promotes in vitro cisplatin resistance and proliferation in non‐small‐cell lung cancer (NSCLC) cells. (A) Levels of miR‐20a expression were assessed in HCC827 and H1299 cells following miR‐20a mimic transfection, miR‐NC mock transfection or control treatment. The viability (B and C) and DDP IC50 values (D) for NSCLC cell lines treated as in (A) were measured. The proliferation (E and F), colony formation (G and H) and Hoechst staining (I and J) results for NSCLC cell lines treated as in (A). (K and L) Western blotting was used to assess PI3K‐p85, PI3K‐p100, PI3K, pAKT and AKT expression in the indicated cell lines in the presence or absence of DDP treatment, with GAPDH as a normalization control. Outcomes are means ± standard deviation (SD) (*p < .05, **p < .01, ***p < .001 vs. control; ^##^ *p* < .01, ^###^ *p* < .001 vs. mock + DDP)

To more fully clarify the mechanisms whereby miR‐20a contributes to proliferation and the induction of chemoresistance in NSCLC cells, we next transfected HCC827 and H1299 cells with miR‐20a mimics, resulting in significant increases in the expression of this miRNA as compared to Mock transfection (Figure 4A). Consistent with the above results, the viability of both of these cell lines in a CCK‐8 assay rose following miR‐20a mimic treatment (Figure 4B,C), with a concomitant increase in DDP IC50 values relative to those for control cells (Figure 4D and Table 2). Overexpressing miR‐20a also increased cellular proliferation in an EdU uptake assay at baseline and upon DDP treatment (Figure 4E,F), and consistent increases in colony formation activity were also observed (Figure 4G,H). When Hoechst staining was implemented for the assessment of the apoptotic death of NSCLC cells, miR‐20a mimic transfection was further found to suppress apoptotic death for both HCC827 and H1299 cells at baseline and upon DDP treatment (Figure 4I,J). Western blotting additionally revealed that miR‐20a mimic treatment resulted in increases in PI3K‐p85, PI3K‐p110 and pAKT expression in these cell lines, while miR‐20a inhibitor treatment yielded the opposite phenotypes (Figure 4K,L). The same was also true in DDP‐treated NSCLC cell lines. Overall, these data thus illuminated that miR‐20a can enhance proliferation and DDP resistance in NSCLC cells.

**TABLE 2:** IC50 of DDP in HCC828 and H1299 cells transfected with miR‐20a mimics

	HCC827 (μg/ml)	H1299 (μg/ml)
Control	4.50 ± .33	5.42 ± .36
Mock	4.04 ± 1.14	5.49 ± .41
miR‐20a mimics	19.82 ± 1.46^***^	22.80 ± 1.60^***^

Abbreviation: DDP, Cisplatin.

^***^ *p* < .001 versus Control.

### 3.5 miR‐20a directly targets PTEN

**FIGURE 5:**
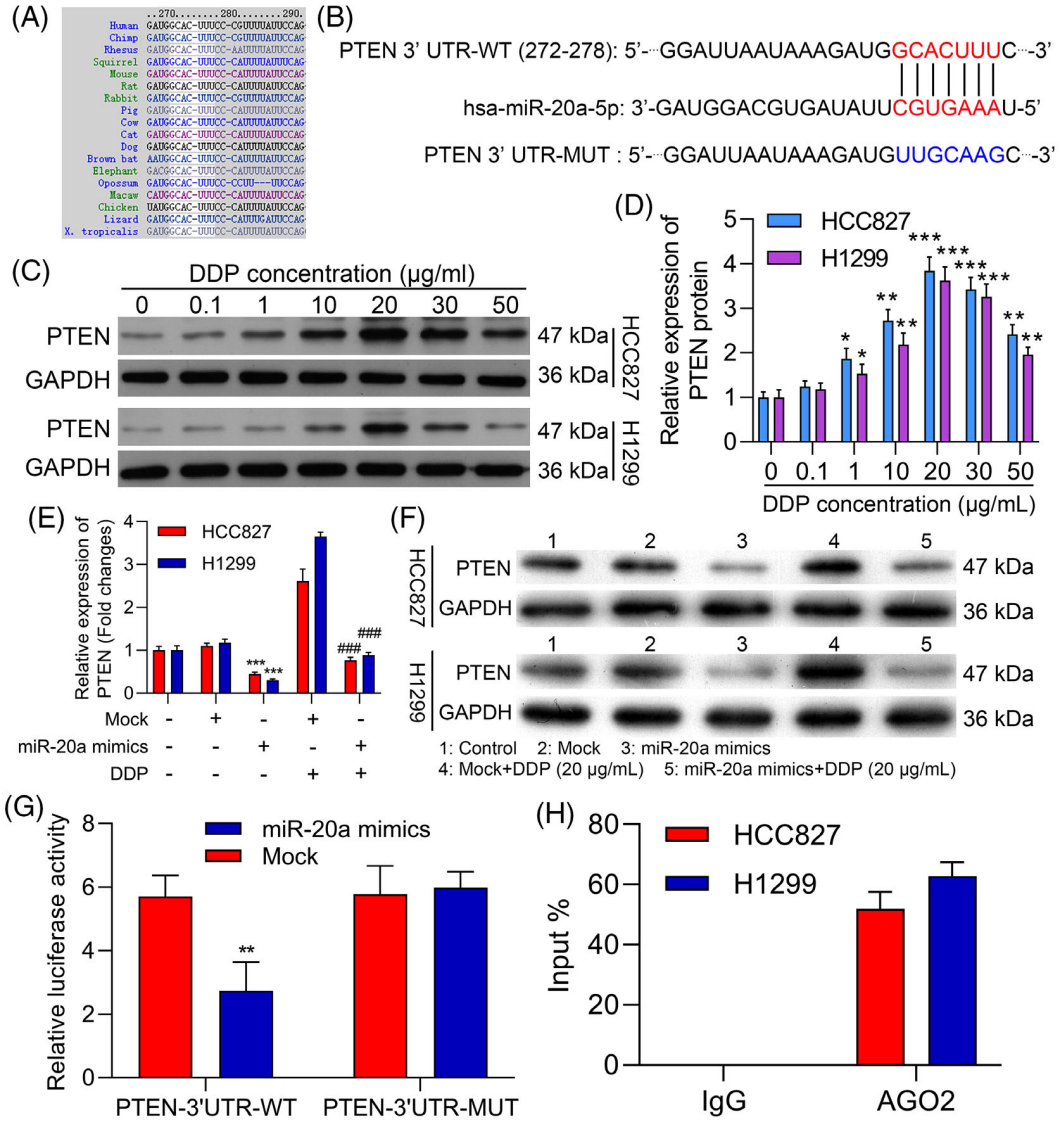
miR‐20a directly targets PTEN. (A) Bioinformatics analyses identified PTEN as a putative miR‐20a target gene. (B) Schematic overview of luciferase reporter constructs with WT or mutant (MUT) PTEN 3′‐UTR binding sites. (C and D) Relative PTEN expression in the indicated cells was measured via Western blotting in the HCC827 and H1299 cells treated with 0, .1, 1, 10, 20, 30, 50 μg/ml DDP. (E and F) HCC827 and H1299 cells were processed with miR‐20a mimics or/and 20 μg/ml DDP, and the level of PTEN protein was identified through Western blot. ***p < .001 versus control; ^###^ *p* < .001 versus mock + DDP. (G) Luciferase activity in HEK‐293T cells was evaluated following co‐transfection of miR‐20a mimics and WT or MUT reporter plasmids. (H) RNA immunoprecipitation was used to detect physical interactions between PTEN and miR‐20a. Results are representative data from triplicate experiments, and outcomes are means ± standard deviation (SD) (**p < .01)

To clarify the mechanisms whereby miR‐20a may influence tumour progression, we next employed a bioinformatics approach to clarify potential targets of this miRNA. We first predicted the possible target genes of miR‐20a through DIANA‐microT, miRanda, PicTar and TargetScan databases. As shown in the Venn diagram, there were 202 target genes that can be predicted in these four databases (Figures S3A). The results of GO analysis denoted that for BP, the 202 target genes were highly enriched in the homophilic cell adhesion and cell‐cell adhesion; for MF, the 202 target genes were mainly enriched in Rho GTPase binding, small GTPase binding, DNA‐binding transcription repressor activity and RNA polymerase II; for CC, they were obviously enriched in apical junction complex, cell–cell junction, probable transcription factor body and cytoplasmic side of plasma membrane (Figures S3B). The results of KEGG analysis indicated that 202 target genes were particularly enriched in endocytosis, regulation of actin cytoskeleton, Rap1 signalling pathway and tight junction, etc (Figure S3C,D). Through differential expression, pathway and literature analysis, PTNE was selected as a potential target of miR‐20a. And, we also discovered that PTEN sequences were homologous in different species (Figure 5A). Consistently, a candidate miR‐20a site of binding was identified within the PTEN 3′‐UTR (Figure 5B). Furthermore, we also found that DDP reduced PTEN protein expression in both HCC827 and H1299 cells (Figure 5C,D). Meanwhile, we further investigated the influence of miR‐20a and DDP on PTEN expression in HCC827 and H1299 cells. The data denoted that overexpression of miR‐20a prominently down‐regulated PTEN, while DDP treatment notably up‐regulated PTEN in HCC827 and H1299 cells; and miR‐20a overexpression and DDP can partially reverse the influence of the other party on PTEN expression in HCC827 and H1299 cells (Figure 5E,F).

To confirm the capability of miR‐20a for direct regulation of PTEN expression, we next constructed luciferase vectors harboring WT or mutated (MUT) versions of the identified PTEN 3′‐UTR sequence. When these plasmids were co‐transfected into cells along with miR‐20a mimics, decreased luciferase activity was detected in the WT group but not the MUT group (Figure 5G). These data thus confirmed the ability of miR‐20a to directly suppress PTEN expression in a sequence‐specific manner. RIP experiments additionally confirmed miR‐20a co‐precipitation with PTEN, consistent with a robust interaction between these two targets (Figure 5H). These results thus validated PTEN as a miR‐20a target gene.

### 3.6 Overexpressing PTEN suppresses NSCLC cell proliferation and chemoresistance while promoting apoptotic death

**FIGURE 6:**
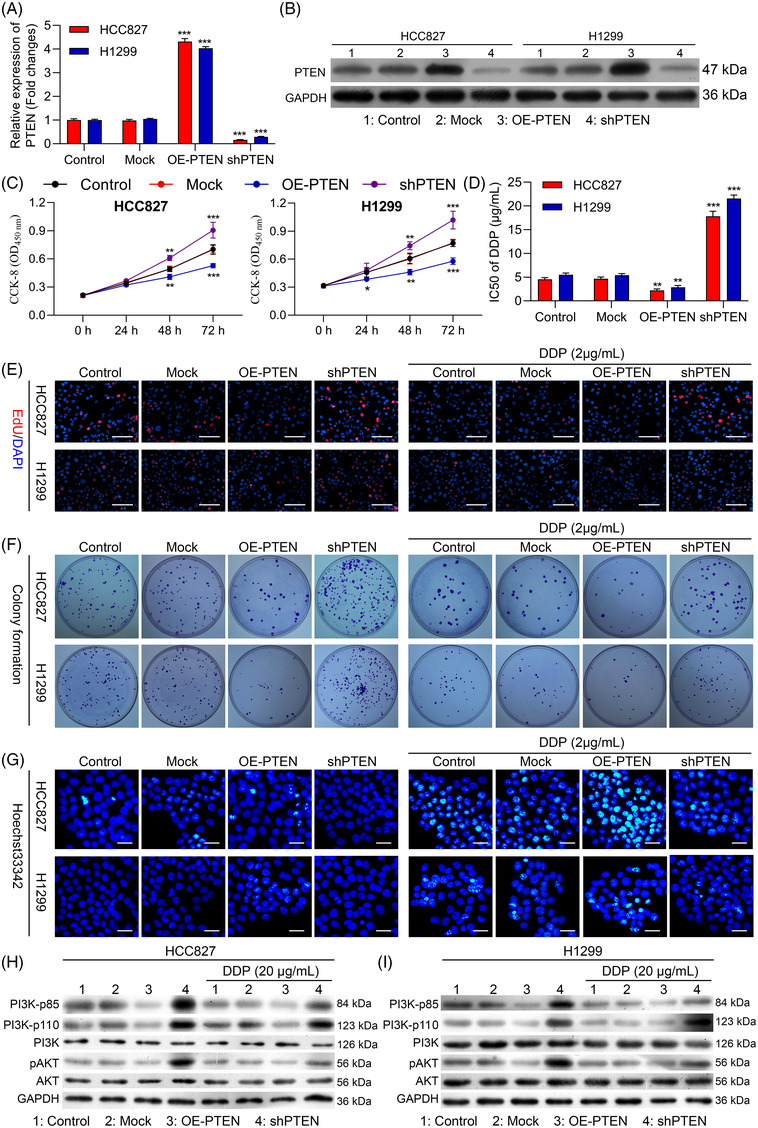
Overexpression of PTEN suppresses non‐small‐cell lung cancer (NSCLC) cell chemoresistance, proliferation and apoptotic resistance. PTEN was knocked down or overexpressed in H1299 and HCC8237 cells. (A and B) PTEN expression in these cells was examined via qPCR and Western blotting. Cellular viability (C) and DDP IC50 values (D) were measured. (E) The proliferation of these cells was monitored via EdU uptake assay in the absence (left) or presence of DDP (2 μg/ml; Right). (F) The proliferation of these cells was monitored via colony formation assay in the absence (left) or presence of DDP (2 μg/ml; Right). (G) The apoptotic ratio values for these cells were measured via Hoechst staining in the absence (left) or presence of DDP (20 μM; Right). (H and I) PI3K‐p85, PI3K‐p100, PI3K, pAKT and AKT levels were measured via Western blotting the indicated cells in the presence or absence of DDP treatment. GAPDH employed as a normalization control. Outcomes are means ± standard deviation (SD) (*p < .05; **p < .01; ***p < .001)

To further clarify the functional importance of PTEN in NSCLC cells, it was next knocked down with an appropriate shRNA construct (shPTEN) or overexpressed with an appropriate plasmid (OE‐PTEN) in HCC827 and H1299 cells, with Western blotting and qPCR being used to confirm successful knockdown or overexpression of this gene at the mRNA and protein levels (Figure 6A,B). No differences in the proliferation of either tested cell line were evident within 24 h in a CCK‐8 assay (*p* > .05), whereas PTEN‐overexpressing cells exhibited impaired proliferation after 48 h (Figure 6C). PTEN overexpression significantly reduced DDP IC50 values for both tested cell lines (Figure 6D and Table 3), and an EdU uptake assay further confirmed that overexpressing improved the ability of HCC827 and H1299 cells to proliferate in with or without of DDP treatment (Figure 6E and Figure S4A), with a colony formation assay yielding similar results (Figure 6F and Figure S4B). Hoechst staining was further applied to appraise the apoptotic death of these cells, revealing that overexpressing PTEN increased the frequency of apoptotic HCC827 and H1299 cells in the with or without of DDP treatment (Figure 6G and Figure S4C).

**TABLE 3:** IC50 of DDP in HCC828 and H1299 cells transfected with PTEN overexpression and knockdown plasmids

	HCC827 (μg/ml)	H1299 (μg/ml)
Control	4.54 ± .37	5.51 ± .37
Mock	4.65 ± .37	5.42 ± .32
OE‐PTEN	2.22 ± .29^**^	2.86 ± .40^**^
shPTEN	17.80 ± 1.08^***^	21.56 ± .75^***^

Abbreviation: DDP, Cisplatin.

^**^ *p* < .01.

^***^ *p* < .001 versus Control.

Western blotting further indicated that PTEN knockdown resulted in significant increases in PI3K‐p85, PI3K‐p110 and pAKT expression in the with or without of DDP treatment (Figure 6H,I). Overexpressing PTEN can thus compromise the proliferative activity, chemoresistance and apoptotic resistance of HCC827 and H1299 cells.

### 3.7 miR‐20a promotes NSCLC cell progression and chemoresistance by suppressing PTEN expression

**FIGURE 7:**
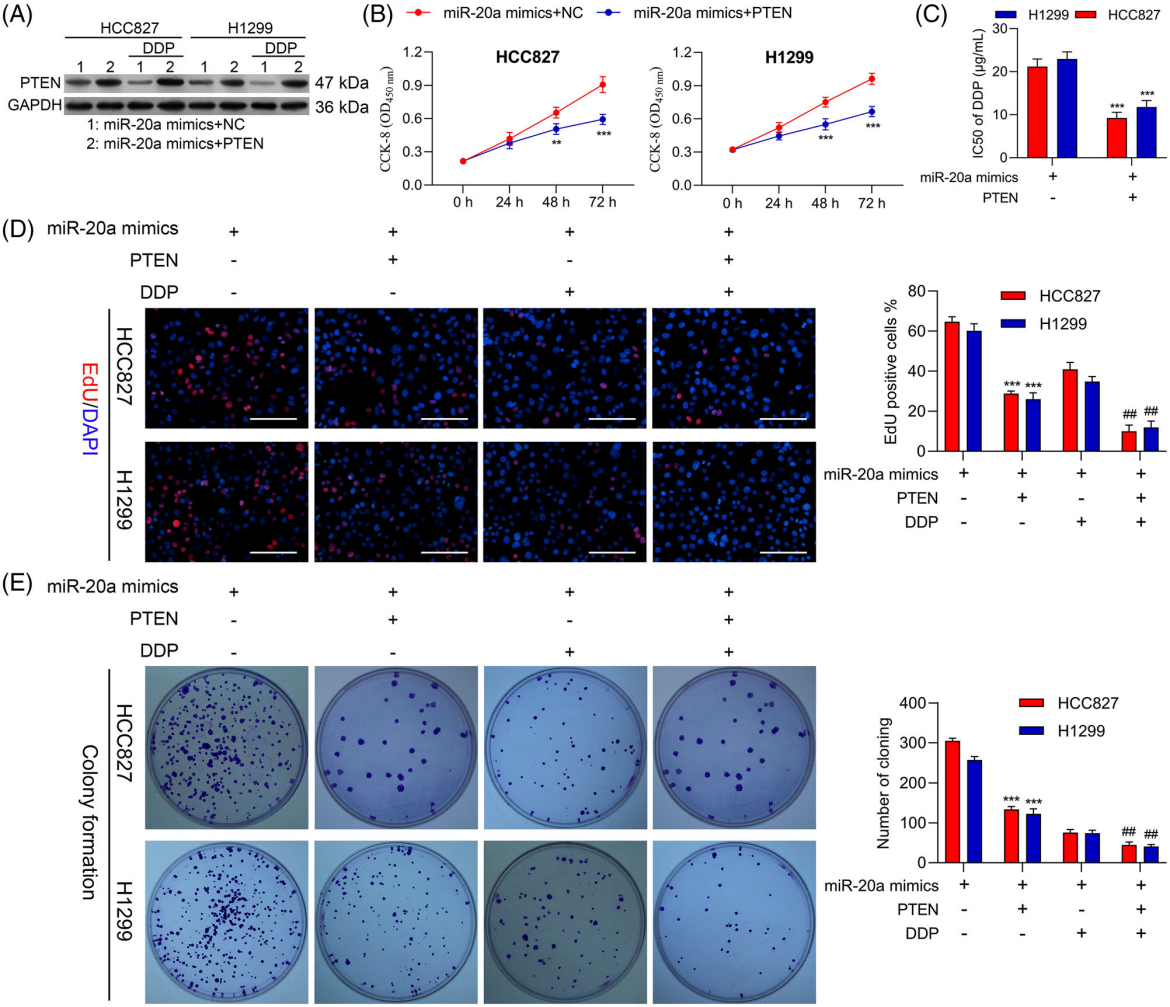
miR‐20a targets PTEN to induce chemoresistance and progression in non‐small‐cell lung cancer (NSCLC) cells. Following miR‐20a mimic transfection and PTEN overexpression (OE‐PTEN) or NC plasmid transfection, cells were analyzed via (A) Western blotting demonstrating that miR‐20a regulates PTEN expression. (B) CCK‐8 assessment was implemented to analyse cell viability. (C) CCK‐8 assessment was implemented to appraise DDP IC50 values for HCC827 and H1299 cells. (D) Cellular proliferation following miR‐20a mimic transfection and/or PTEN overexpression was assessed via EdU uptake assay. (E) A colony formation assessment was employed to appraise H1299 and HCC827 cell proliferation. Results are representative data from triplicate experiments, and outcomes are means ± standard deviation (SD) (**^,##^ *p* < .01; ***p < .001)

To evaluate the degree to which miR‐20a‐mediated PTEN down‐regulation enhances NSCLC tumour growth, we subsequently executed a series of rescue assessments. When NSCLC cells were co‐transfected with miR‐20a mimics and OE‐PTEN, PTEN was up‐regulated relative to the levels observed in cells with miR‐20a mimics and negative control (NC) plasmids co‐transfection in both the presence and absence of DDP treatment (Figure 7A). CCK‐8 assessments illustrated that the overexpression of PTEN suppressed miR‐20a‐induced proliferative activity in both HCC827 and H1299 cells (Figure 7B), with a similar reduction in the DDP IC50 values for both of these tested cell lines following miR‐20a mimic and OE‐PTEN co‐transfection relative to Mock transfection (Figure 7C and Table 4).

**TABLE 4:** IC50 of DDP in HCC828 and H1299 cells co‐transfected with miR‐20a mimics and PTEN overexpression plasmids

	HCC827 (μg/ml)	H1299 (μg/ml)
miR‐20a mimics	21.19 ± 1.76	22.94 ± 1.66
miR‐20a mimics+OE‐PTEN	9.26 ± 1.29^***^	11.80 ± 1.49^***^

Abbreviation: DDP, Cisplatin.

***p < .001.

We further evaluated the influence of miR‐20a and PTEN on the multiplication and chemosensitivity of NSCLC cells by simultaneously co‐transfecting H1299 and HCC827 cells with miR‐20a mimics and either OE‐PTEN or NC plasmids, after which cells were subjected to DDP treatment. PTEN overexpression impaired NSCLC cell proliferation and colony formation activity in this assay system (Figure 7D,E), with miR‐20a and OE‐PTEN co‐transfection resulting in significantly enhanced DDP sensitivity relative to mock transfection. Apoptosis assays further revealed that PTEN was able to suppress tumour growth and chemoresistance through interactions with miR‐20a in both tested cell lines (Figure S5A). These data further supported the conclusion that PTEN is a direct miR‐20a target gene with tumour suppressor functionality in NSCLC.

Western blotting further revealed that OE‐PTEN and miR‐20a mimic co‐transfection suppressed PI3K‐p85, PI3K‐p110 and pAKT levels at baseline and in the context of DDP treatment (Figures S5B). Conclusively, these above findings explain that PTEN is an essential miR‐20a target linked with the ability of this miRNA to promote NSCLC cell growth and chemoresistance.

### 3.8 miR‐20a‐containing exosomes derived from CAFs modulate the PI3K/AKT pathway to promote in vivo NSCLC cell tumour growth and chemoresistance

**FIGURE 8:**
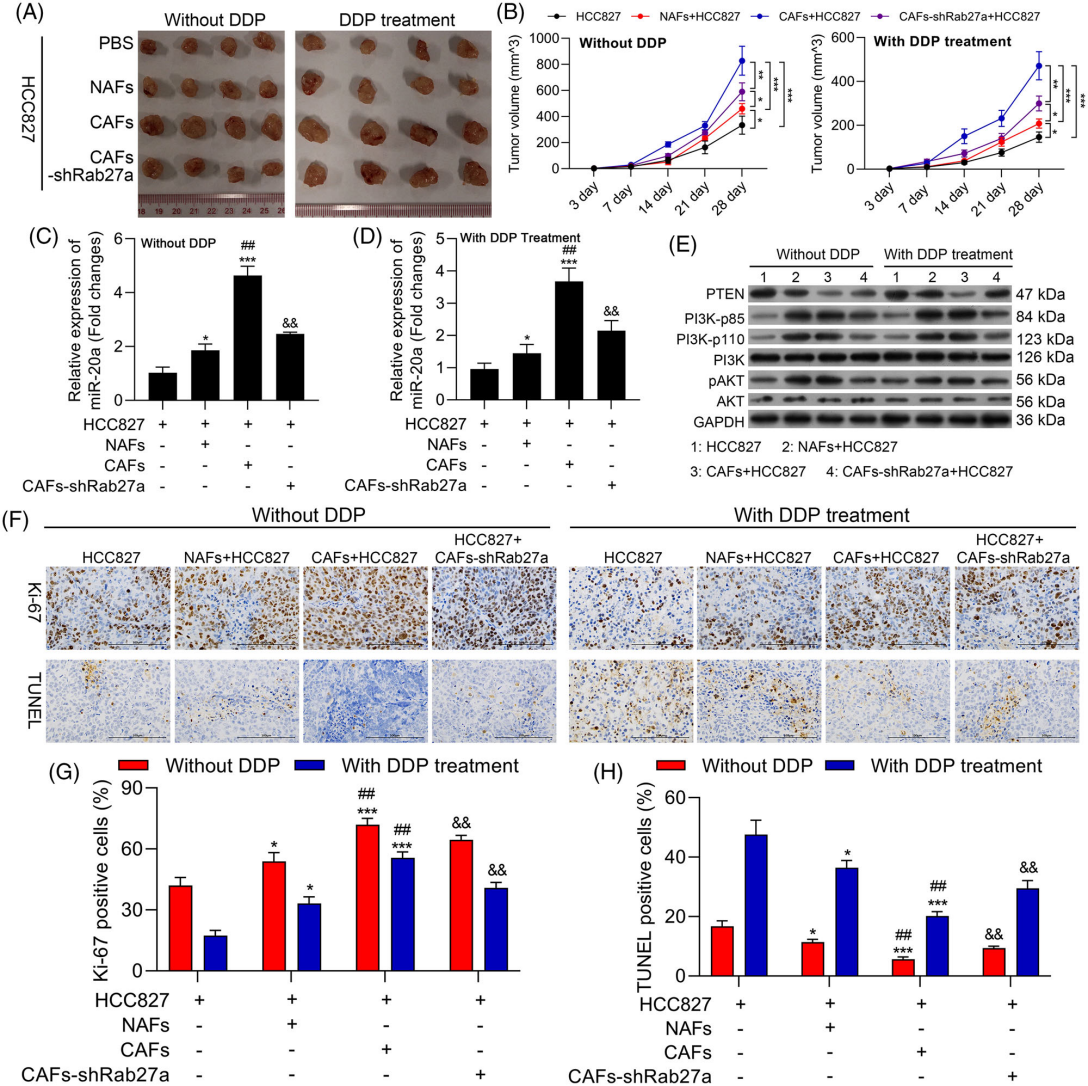
miR‐20a‐containing exosomes derived from cancer‐associated fibroblasts (CAFs) regulate the PI3K/AKT pathway in vivo to promote non‐small‐cell lung cancer (NSCLC) tumour growth and chemoresistance. Nude mice were subcutaneously implanted with HCC827 cells treated using PBS, normal tissue‐associated fibroblasts (NAFs), CAF or CAFs‐shRab27a. Mice were then randomized into DDP treatment and untreated control groups. (A) Images of representative xenograft tumours. (B) Tumour volumes were analysed once weekly beginning 1 week after implantation. (C) The expression of miR‐20a was assessed via qPCR in tumours from untreated mice. (D) The expression of miR‐20a was assessed via qPCR in tumours from DDP‐treated mice. (E) PTEN, PI3K‐p85, PI3K‐p100, PI3K, pAKT and AKT were analysed via Western blotting in tumour‐bearing mice. (F) Tumours from xenograft model mice were subjected to Ki‐67 and TUNEL staining to analyse apoptotic cell death (200x), with the positive staining area being quantified in (G and H). Results are representative data from triplicate experiments, and outcomes are means ± standard deviation (SD) (*p < .05; **p < .01; ***p < .001)

Finally, we sought to expand the above results to an in vivo model system by subcutaneously implanting nude mice with HCC827 tumour cells that had been mixed with NAFs, CAFs or CAFs‐shRab27a. Those tumours that were co‐transplanted with CAFs exhibited significantly enhanced growth relative to those co‐transplanted with NAFs, while CAFs‐shRab27a co‐transplantation significantly reduced tumour growth and reversed the observed changes in DDP sensitivity (Figure 8A,B). Subsequent qPCR analyses indicated that miR‐20a expression levels were evident in the HCC827/CAF group relative to the HCC827/CAF‐shRab27a group in both the presence and absence of DDP treatment (Figure 8C,D). These data indicate that CAFs can influence the composition of xenograft tumours in vivo, with miR‐20a enhancing the multiplication and DDP resistance of NSCLC cells in this context. Western blotting further indicated that HCC827/CAF implantation was associated with down‐regulated PTEN, up‐regulated PI3K‐p85, PI3K‐p110 and pAKT, whereas the opposite was observed in the HCC827/CAF‐shRab27a group in the with or without of DDP treatment (Figure 8E). Moreover, we observed increased intratumoural Ki‐67 expression in the HCC827/CAF group relative to the HCC827/NAF and HCC827/CAF‐shRab27a groups, with the same also being true following cisplatin treatment (Figure 8F,G). TUNEL staining was performed to evaluate apoptotic cell death within xenograft tumours, revealing significant reductions in intratumoural apoptosis in the HCC827/CAF group relative to the HCC827/NAF group, while apoptosis rates were increased in the HCC827/CAF‐shRab27a group. The same was also observed following DDP treatment, with CAFs suppressing the ability of this chemotherapeutic agent to inhibit apoptotic cell death (Figure 8F,H), suggesting that miR‐20a can significantly reduce the chemotherapeutic efficacy of DDP. Overall, these findings explain that miR‐20a‐enriched exosomes derived from CAFs were able to down‐regulate PTEN and thereby regulate the PI3K/AKT pathway in vivo to regulate NSCLC tumour chemoresistance and growth.

### 3.9 Exosomes derived from CAFs induce tumour growth and chemoresistance and activate PI3K/AKT pathway in a nude mouse NSCLC model

**FIGURE 9:**
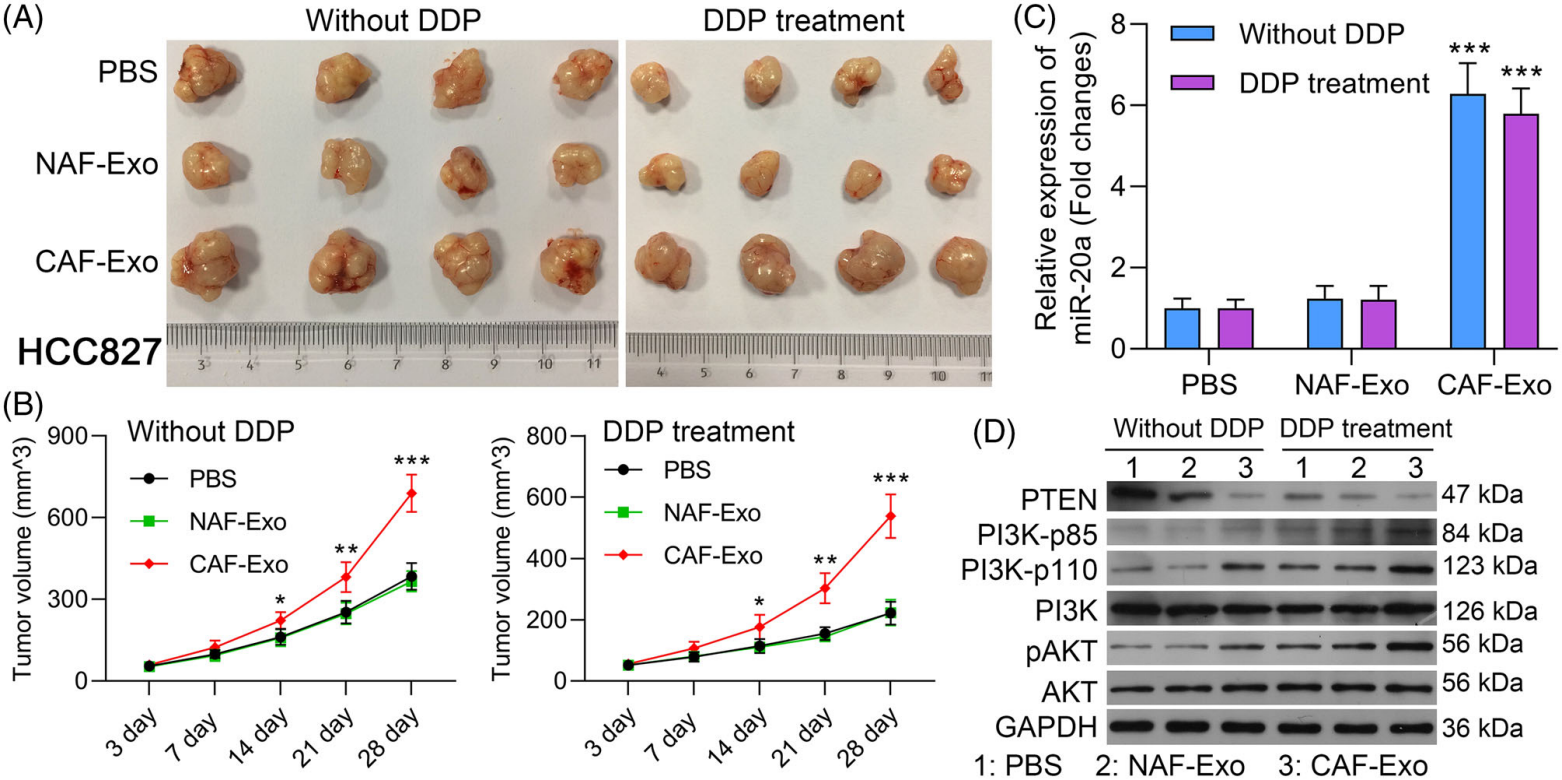
Exosomes derived from cancer‐associated fibroblasts (CAFs) induce tumour growth and chemoresistance, and activate PI3K/AKT pathway in a nude mouse non‐small‐cell lung cancer (NSCLC) model. HCC827 cells were processed with exosomes derived from normal tissue‐associated fibroblasts (NAFs) or CAFs were subcutaneously injected into nude mice, which were treated or untreated with DDP. (A) Subcutaneous tumours in each group were excised and displayed. (B) Tumour volume was measured at the end of 3, 7, 14, 21 and 28 days. (C) qPCR analysis of miR‐20a expression in tumours. (D) Western blotting analysis of PTEN, PI3K‐p85, PI3K‐p100, PI3K, pAKT and AKT. Outcomes are means ± standard deviation (SD) (*p < .05; **p < .01; ***p < .001)

Based on the research results in Figure 8, we further explore the impacts of exosomes derived from CAFs on NSCLC growth and chemoresistance to exclude other vesicles or cell to cell communication methods mediated miR‐20a into tumour cells. We first discovered that relative to exosomes derived from NAFs, exosomes derived from CAFs markedly increased the tumour volume of mice, which basically consistent with that of CAFs (Figure 9A,B). Besides, the data uncovered that exosomes derived from CAFs could dramatically up‐regulate miR‐20a in DDP treated and untreated mouse tumour tissue (Figure 9C). Moreover, Western blotting data disclosed that exosomes derived from CAFs could reduce PTEN expression, increase PI3K‐p85, PI3K‐p110 and pAKT expressions in DDP treated and untreated mouse tumour tissue (Figure 9D). Overall, this part of the data further revealed that exosomes derived from CAFs, which is basically consistent with CAFs, also could enhance tumour growth and chemoresistance of NSCLC cells, and activate PI3K/AKT pathway in vivo.

### 3.10 Overexpression of miR‐20a promotes cisplatin‐resistant

**FIGURE 10:**
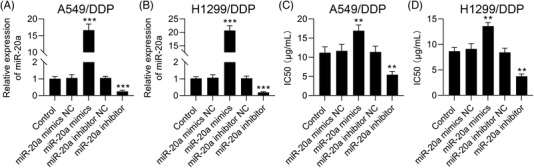
Overexpression of miR‐20a promotes cisplatin‐resistant in A549/DDP and H1299/DDP cells. (A and B) qRT‐PCR results showed that the expression levels of miR‐20a were increased or decreased in A549/DDP and H1299/DDP cells after miR‐20a mimics or inhibitor transfection. (C and D) Overexpression of miR‐20a led to a substantial increase in the DDP IC50 value, whereas suppression of miR‐20a expression by miR‐20a led to a substantial decrease in the DDP IC50 value in A549/DDP and H1299/DDP cells, and outcomes are means ± standard deviation (SD) (**p < .01; ***p < .001)

To further determine the role of miR‐20a in chemoresistance of NSCLC, cisplatin‐resistant cell lines with miR‐20a mimics and inhibitor transfection were used to measure the role of miR‐20a on the chemosensitivity of cisplatin‐resistant cells to DDP. First, miR‐20a expressions were increased or decreased in cisplatin‐resistant cells after miR‐20a mimics or inhibitor transfection (Figure 10A,B). Furthermore, overexpression of miR‐20a by miR‐20a mimics transfection led to a substantial increase in the DDP IC50 value, whereas repression of miR‐20a expression levels via miR‐20a inhibitor transfection led to a substantial decrease in the DDP IC50 value in cisplatin‐resistant cells (Figure 10C,D). The results confirmed that miR‐20a participates in chemoresistance of NSCLC.

### 3.11 Expression levels of miR‐20a and PTEN in NSCLC tissues

**FIGURE 11:**
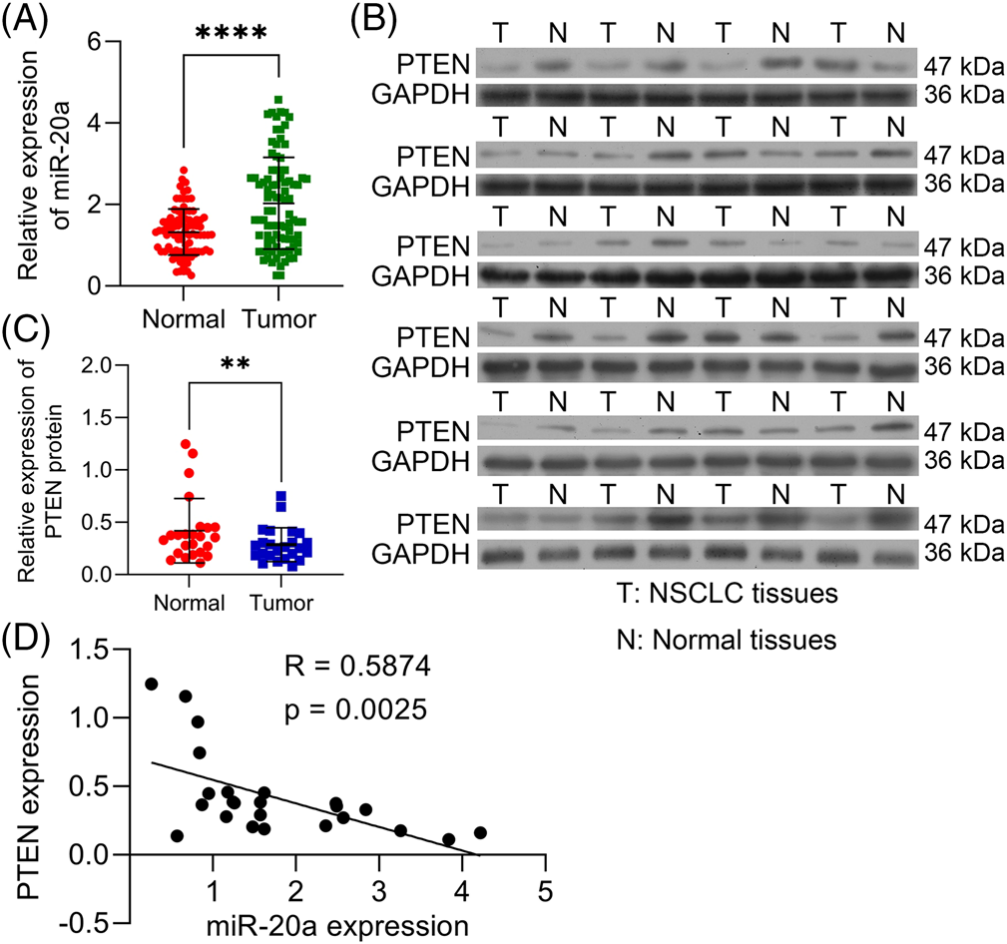
PTEN is down‐regulated and is negatively correlated with miR‐20a expression in non‐small‐cell lung cancer (NSCLC) tissues. (A) qPCR results showed that miR‐20a was remarkably increased in NSCLC tissues compared with tumour‐adjacent normal tissues (*n* = 90). (B and C) Western blot results showed that PTEN was significantly decreased in cancer tissues (*n* = 24). (D) The expression levels of PTEN were negatively correlated with miR‐20a expression levels (*n* = 24). Outcomes are means ± standard deviation (SD) (**p < .01; ****p < .0001)

To further confirm the influence of miR‐20a in NSCLC, we evaluated miR‐20a expression levels in 90 pairs of NSCLC tissues and tumour‐adjacent normal tissue. Here, we found that miR‐20a was remarkably aggrandized in NSCLC tissues (*n* = 90) (*p* < .0001, Figure 11A). To inspect the relevance between expression levels of miR‐20a and the clinical and pathological features in NSCLC patients, patients with NSCLC were divided into two groups (high and low group) on the basis of median values (cutoff = 1.80) of miR‐20a expression. Here, our findings showed that miR‐20a expression levels were noticeably associated with tumour differentiation (*p* = .0061), lymph node metastasis (*p* = .0003) and tumor node metastasis (TNM) stage (*p* < .0001) (Table 5). To confirm the above results, the protein expressions of PTEN in NSCLC tissues and pericarcinomatous tissues were detected using Western blot assay, the results revealed that PTEN was observably reduced in cancer tissues (Figure 11B,C), and the expression levels of PTEN were negatively correlated with miR‐20a expression levels (Figure 11D). Therefore, miR‐20a‐PTEN axis represents a potential target for predicting the progression of NSCLC patients.

**TABLE 5:** Correlation between miR‐20a expression and clinicopathological features in 90 cases of non‐small‐cell lung cancer (NSCLC)

		miR‐20a expression (*n* = 90)		
Factors	*N*	Low expression (*n* = 45)	High expression (*n* = 45)	*p‐*Value
**Age**				
<60	50	23	27	.3961
≥60	40	22	18	
**Gender**				
Male	48	23	25	.6726
Female	42	22	20	
**TNM stage**				
I ‐ II	49	35	14	**<.0001**
III ‐ IV	41	10	31	
**Differentiation**				
Poor	47	17	30	**.0061**
Well	43	28	15	
**Lymph node metastasis**				
Yes	41	12	29	**.0003**
No	49	33	16	
**Smoking status**				
Smoker	51	27	24	.5234
Nonsmoker	39	18	21	

## 4 DISCUSSION

Patients with NSCLC often exhibit a poor prognosis owing to rapid tumour growth and the emergence of chemoresistance.^2^ While early‐stage NSCLC is generally treated via surgery, the appreciable number of patients is primarily diagnosed with metastatic or locally advanced disease and thus receives platinum‐based cisplatin as a standard treatment.^52^, ^53^ However, the acquisition of cisplatin resistance ultimately results in treatment failure and poor patient outcomes.^54^, ^55^ As such, there is an obvious need to more fully discover the strategies governing NSCLC cell malignancy and cisplatin resistance in an effort to guide better patient treatment.

Recent work suggests that the TME is a key determinant of tumour chemoresistance.^56^, ^57^ CAFs are the most plentiful cells within the TME.^9^ Studies confirmed that the high heterogeneity of CAFs allows them to exhibit a dual function in disease: on the one hand, CAFs can induce angiogenesis, inflammation, immunosuppression and metastasis, ultimately leading to tumour progression; on the other hand, the anti‐tumour properties of fibroblasts regulate processes such as extracellular matrix production, epithelial cell growth and differentiation and response to tissue injury.^58^, ^59^ Besides, CAFs are also active mediators of tumour response to chemotherapy.^60^ In prior reports, lung cancer cell‐derived CAFs are highly resistant to DDP treatment, with CM derived from these cells enhancing the growth and DDP resistance of lung cancer cells.^25^ Lung cancer cells also exhibit improved DDP resistance when cultured together with CAFs. We therefore concluded that NSCLC cells were able to render NSCLC cells refractory to chemotherapeutic treatment through mechanisms that have yet to be fully clarified. Herein, we confirmed that CAFs are key regulators of NSCLC growth and therapeutic resistance. Studies have also revealed that CAFs can transduce some growth factors and cytokines by controlling autocrine or paracrine signals and can secrete exosomes to carry and transport ncRNAs, proteins, DNA and other substances, which in turn can communicate with cancer cells on an intercellular basis.^13^, ^61^ Thus, we speculated that the role of CAFs in NSCLC might be through mechanisms tied to exosome‐mediated intracellular communication. A range of cell types secrete exosomes, which can thereupon modulate key processes including invasion, angiogenesis and metastasis.^15^, ^62^ CAF‐derived exosomes were able to enhance breast cancer cell proliferation and to induce the epithelial‐mesenchymal transition via the regulation of the CDX2/HOXA5 pathway.^63^ The exosome‐mediated transfer of tumour‐associated macrophage‐derived miR‐20a has similarly been shown to promote gastric cancer cell resistance to DDP.^23^ The ability of these exosomes to regulate the aggressiveness of tumour cell growth, however, has yet to be established. Herein, we found that CAF‐derived exosomes were able to promote NSCLC cellular proliferation and chemoresistance through the regulation of the PTEN/PI3K‐AKT signalling axis.

A growing body of evidence indicates that miRNAs could be loaded within exosomes and thereby delivered to recipient cells in which they are able to regulate the expression of genes at the post‐transcriptional level through binding to complementary 3′‐UTR sequences within specific mRNAs.^64^, ^65^ In one prior report, tamoxifen‐resistant breast cancer cell‐derived exosomes were sufficient to promote miR‐221/222 up‐regulation and consequent tamoxifen chemoresistance in recipient endoplasmic reticulum (ER)‐positive breast cancer cells.^29^ Previous investigation suggests that miR‐20a is an essential regulator of immunity, angiogenesis, organ development, and the occurrence of various cancers including breast cancer and liposarcomas.^66^, ^67^, ^68^ The up‐regulation of miR‐20a in NSCLC patients is associated with a worse prognosis and with higher levels of angiogenic activity.^69^ The mechanistic basis for miR‐20a up‐regulation in NSCLC cells, however, remains to be clarified. Herein, we found miR‐20a to be expressed at high levels in CAFs and CAF‐derived exosomes. These exosomes were readily taken up through NSCLC cells, resulting in higher levels of miR‐20a expression within these cells. This CAF exosome‐derived miR‐20a enhanced the apoptosis and chemoresistance of recipient NSCLC cells. In our study, we only adopted shRab27a to inhibit secretion of exosomes in animal experiments. And the reason for not intervening with GW4869 injection is that GW4869 is non‐specific.^70^ GW4869 injection could not directly interfere with CAFs exosome release.

We further confirmed that both PTEN and the associated PI3K/AKT pathway were targets downstream of miR‐20a within NSCLC cells. This signalling axis performs a pivotal task in key processes including the differentiation and renewal of stem cell populations as well as oncogenesis.^71^, ^72^, ^73^ Aberrant signalling of PI3K/AKT has been illustrated to be present within the majority of NSCLC tumours,^74^, ^75^ with several reports having implicated this pathway in the onset and progression of this cancer type.^76^ Herein, we found that CAFs were able to promote aggressive NSCLC cell growth via transferring exosomal miR‐20a to these cells. Overexpressing miR‐20a within NSCLC cells suppressed PTEN expression and thereby increased AKT expression and activation, with CAF‐derived exosomes similarly activating this PI3K/AKT pathway. We thus concluded that miR‐20a‐containing exosomes derived from CAFs may offer value as a prognostic indicator and predictor of chemoresistant status in patients with NSCLC. However, further large‐scale clinical studies will be necessary to validate this model and to explore the potential roles of other CAF‐derived exosomal miRNAs in the context of NSCLC cell chemoresistance.

**FIGURE 12:**
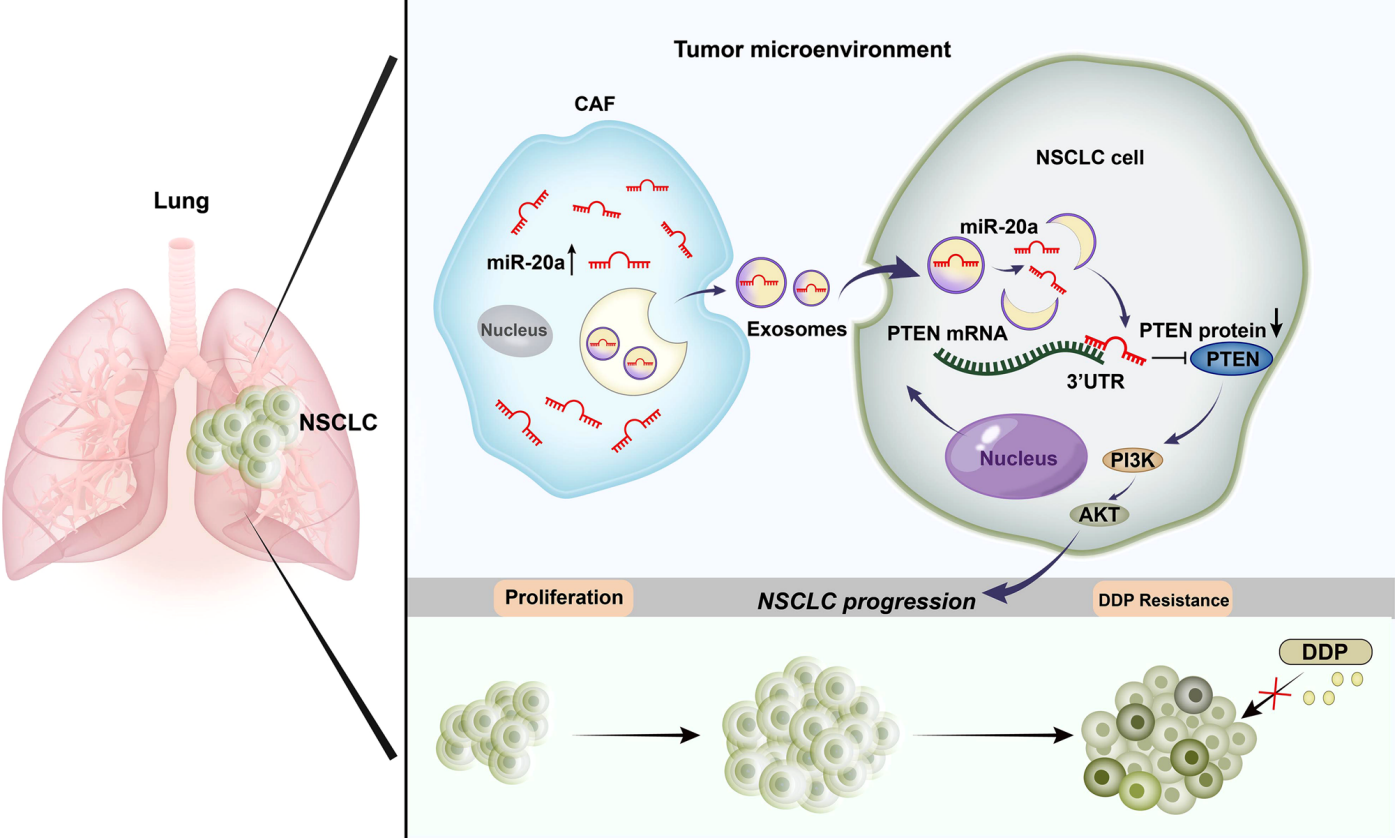
The schematic overview illustrates the mechanistic basis for the observational study results. Cancer‐associated fibroblast (CAF)‐derived miR‐20a is transmitted into non‐small‐cell lung cancer (NSCLC) cells through exosomes and can therein modulate the PTEN/PI3K‐AKT signalling axis to influence tumour growth and chemoresistance

Briefly, the achievements of the current research represent that CAF‐derived exosomes containing miR‐20a can promote chemoresistance and aggressive growth in recipient NSCLC tumour cells owing to the ability of this miRNA to modulate the PTEN/PI3K‐AKT signalling pathway (Figure 12). In conclusion, the obtained data illuminate the inhibition of exosomal miR‐20a as a novel technique to inhibiting NSCLC tumour growth and cisplatin resistance. Our current study focused on NSCLC cell proliferation and chemotherapy resistance and did not explore cell metastasis. And the influence of CAFs‐derived exosomal miR‐20a on NSCLC cell metastasis needs to be further elaborated.

## CONFLICT OF INTEREST

The authors have no relevant financial or non‐financial interests to disclose.

## Supporting information



Supporting Information
