# Supplementary material for: Cancer‐associated fibroblast‐derived exosomal microRNA‐20a suppresses the PTEN/PI3K‐AKT pathway to promote the progression and chemoresistance of non‐small cell lung cancer
Source: Clin Transl Med. 2022 Jul 20;12(7):e989. doi: 10.1002/ctm2.989 (PMC9299573; doi:10.1002/ctm2.989)
Supplement: Supplementary file 1 — Supporting Information [file CTM2-12-e989-s001.docx]

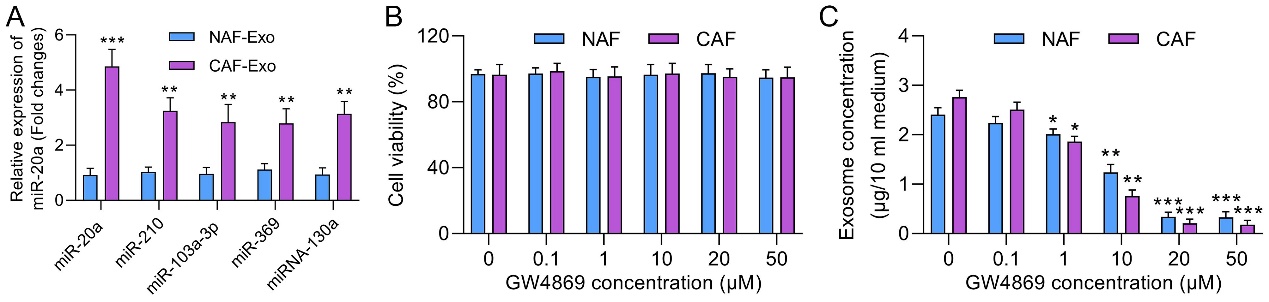


**Figure S1.** **Screening of miRNAs and GW4869 concentration.** (**A**) The levels of miR-20a, miR-210, miR-103a-3p, miR-369, and miR-130a were confirmed using qPCR assay in CAF-and NAF-derived exosomes. (**B**) CAFs and NAFs were processed with 0, 0.1, 1, 10, 20, and 50 μM GW4869, and cell viability was assessed using CCK-8. (**C**) The concentration of exosomes in the medium was examined in CAFs and NAFs, which were treated with different concentrations of GW4869. Outcomes are means ± SEM. (* P < 0.05; ** P < 0.01; *** P < 0.001).


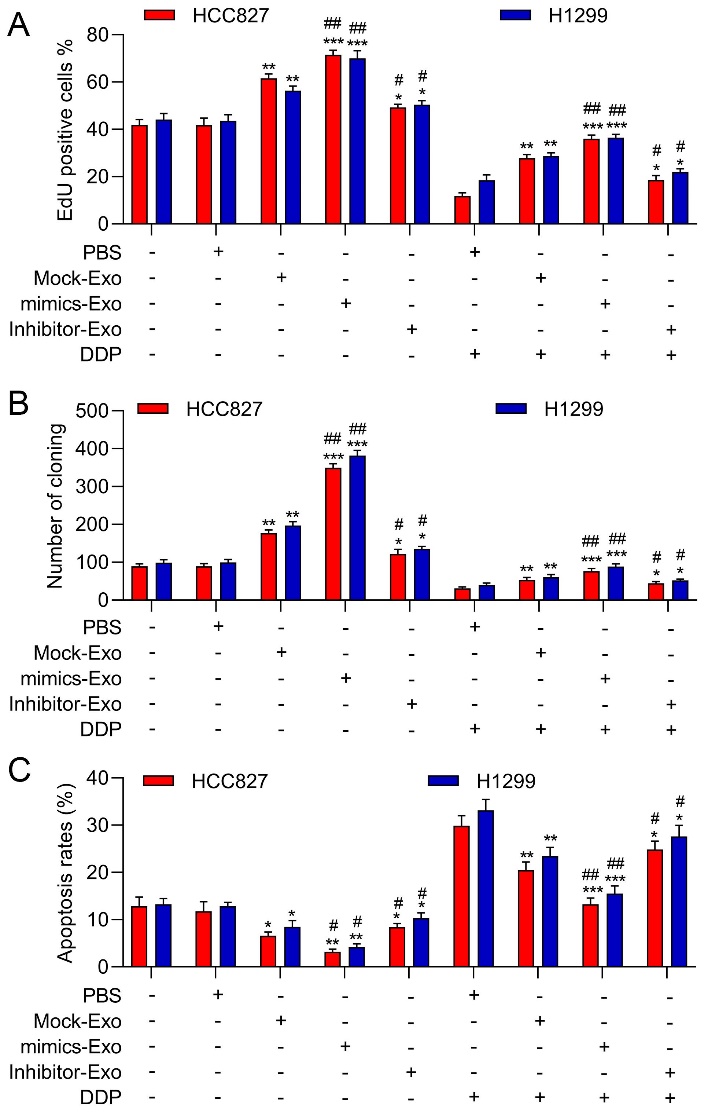


**Figure S2.** The proliferation, apoptotic death, and DDP resistance of H1299 and HCC827 cells was assessed via EdU uptake **(A)**, colony formation **(B)**, and Hoechst assays **(C)**. Images are representative of triplicate assessments, and outcomes are means ± SEM (* P < 0.05, **P < 0.01, ***P < 0.001 vs. PBS; # P < 0.05, ## P < 0.01 vs. Mock-Exosome).


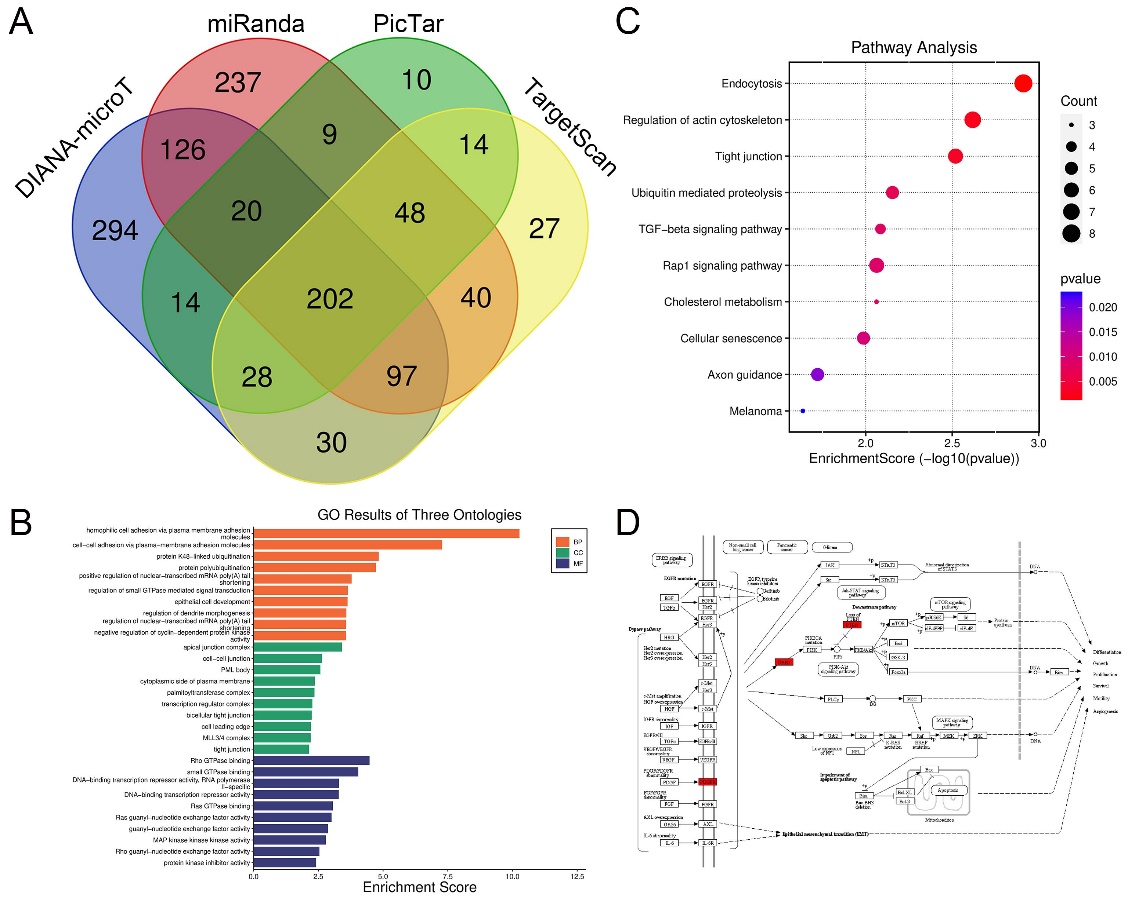


**Figure S3.** **Target gene analysis of miR-20a.** **(A)** Venn diagram analysis of miR-20a target genes based on DIANA-microT, miRanda, PicTar, and TargetScan. **(B)** Go analysis of the 202 target genes related to miR-20a. **(C, D)** Pathway analysis of the 202 target genes related to miR-20a.


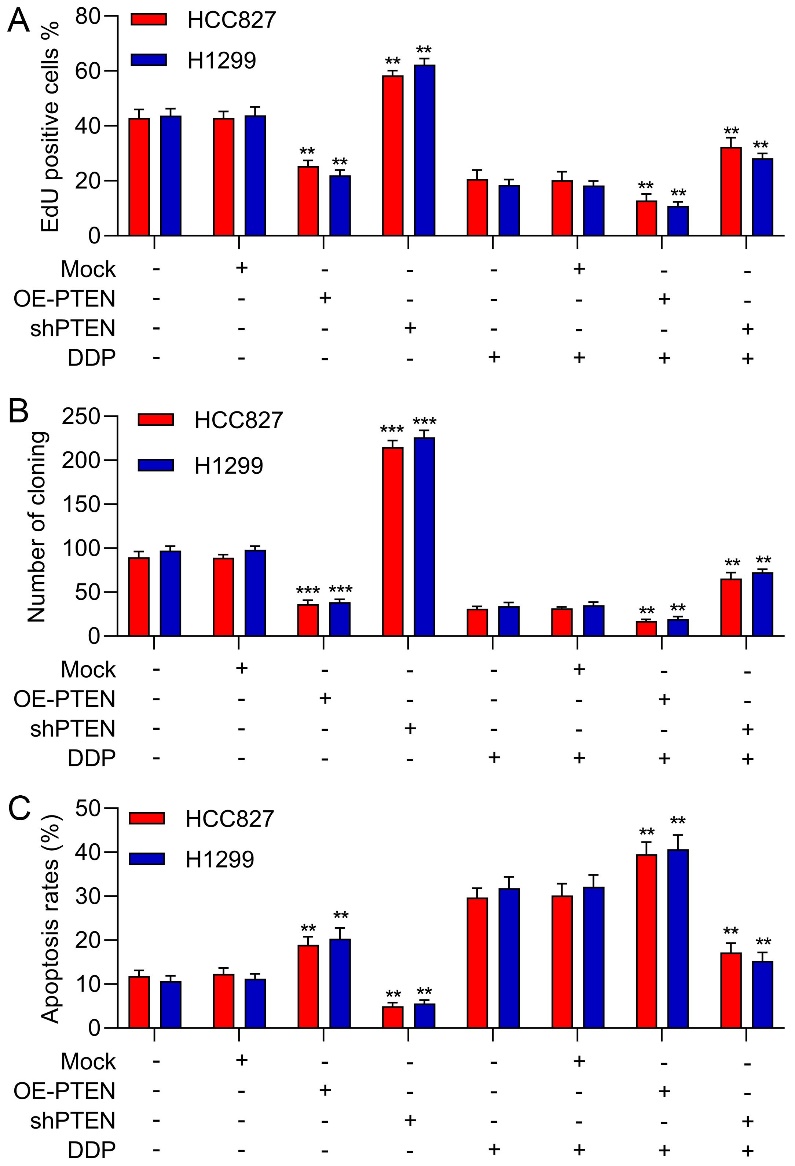


**Figure S4.** **(A)** Quantification of HCC827 and H1299 cellular proliferation based on EdU uptake. **(B)** Quantification of HCC827 and H1299 cellular proliferation based on a colony formation assay. **(C)** Hoechst staining-based analysis of the apoptotic ratio for HCC827 and H1299 cells.


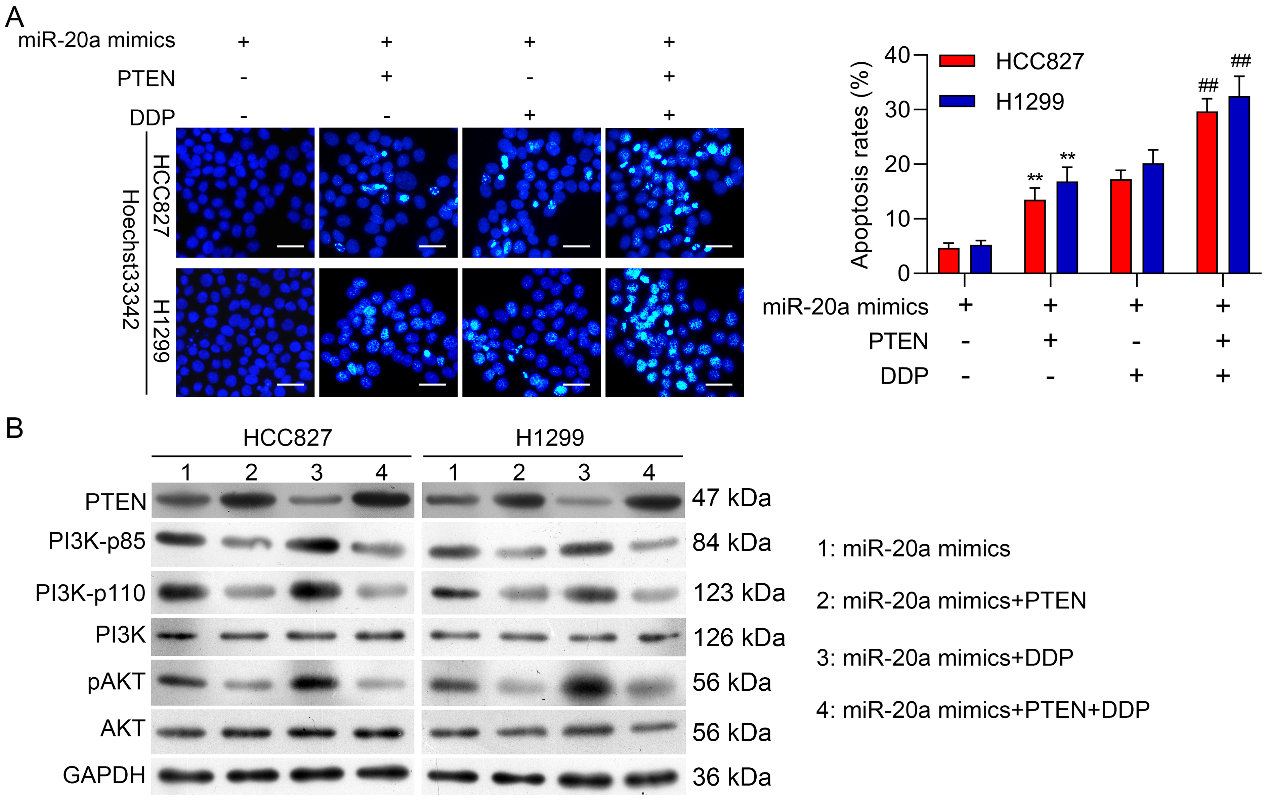


**Figure S5.** Following PTEN overexpression (OE-PTEN) or NC plasmid treatment and miR-20a mimic transfection, HCC827 and H1299 cells were analyzed via **(A)** Hoechst staining to evaluate cellular apoptosis, or **(B)** Western blotting to analyze the expression PI3K-p85, PI3K-p100, PI3K, pAKT, and AKT in the absence or presence of DDP treatment, with GAPDH serving as a normalization control. Outcomes are means ± SEM (** P < 0.01 vs. miR-20a mimic; ## P < 0.01 vs. miR-20a mimic+DDP).
